# ADEMA: An Algorithm to Determine Expected Metabolite Level Alterations Using Mutual Information

**DOI:** 10.1371/journal.pcbi.1002859

**Published:** 2013-01-17

**Authors:** A. Ercument Cicek, Ilya Bederman, Leigh Henderson, Mitchell L. Drumm, Gultekin Ozsoyoglu

**Affiliations:** 1 Department of Electrical Engineering and Computer Science, Case Western Reserve University, Cleveland, Ohio, United States of America; 2 Department of Pediatrics, Case Western Reserve University, Cleveland, Ohio, United States of America; 3 Department of Genetics and Genomic Sciences, Case Western Reserve University, Cleveland, Ohio, United States of America; University of Virginia, United States of America

## Abstract

Metabolomics is a relatively new “omics” platform, which analyzes a discrete set of metabolites detected in bio-fluids or tissue samples of organisms. It has been used in a diverse array of studies to detect biomarkers and to determine activity rates for pathways based on changes due to disease or drugs. Recent improvements in analytical methodology and large sample throughput allow for creation of large datasets of metabolites that reflect changes in metabolic dynamics due to disease or a perturbation in the metabolic network. However, current methods of comprehensive analyses of large metabolic datasets (metabolomics) are limited, unlike other “omics” approaches where complex techniques for analyzing coexpression/coregulation of multiple variables are applied. This paper discusses the shortcomings of current metabolomics data analysis techniques, and proposes a new multivariate technique (ADEMA) based on mutual information to identify expected metabolite level changes with respect to a specific condition. We show that ADEMA better predicts De Novo Lipogenesis pathway metabolite level changes in samples with Cystic Fibrosis (CF) than prediction based on the significance of individual metabolite level changes. We also applied ADEMA's classification scheme on three different cohorts of CF and wildtype mice. ADEMA was able to predict whether an unknown mouse has a CF or a wildtype genotype with 1.0, 0.84, and 0.9 accuracy for each respective dataset. ADEMA results had up to 31% higher accuracy as compared to other classification algorithms. In conclusion, ADEMA advances the state-of-the-art in metabolomics analysis, by providing accurate and interpretable classification results.

## Author Summary

Metabolomics is an experimental approach that analyzes differences in metabolite levels detected in experimental samples. It has been used in the literature to understand the changes in metabolism with respect to diseases or drugs. Unlike transcriptomics or proteomics, which analyze gene and protein expression levels respectively, the techniques that consider co-regulation of multiple metabolites are quite limited. In this paper, we propose a novel technique, called ADEMA, which computes the expected level changes for each metabolite with respect to a given condition. ADEMA considers multiple metabolites at the same time and is mutual information (MI)-based. We show that ADEMA predicts metabolite level changes for young mice with Cystic Fibrosis (CF) better than significance testing that considers one metabolite at a time. Using three different datasets that contain CF and wild-type (WT) mice, we show that ADEMA can classify an individual as being CF or WT based on the metabolic profiles (with 1.0, 0.84, and 0.9 accuracy, respectively). Compared to other well-known classification algorithms, ADEMA's accuracy is higher by up to 31%.

## Introduction

Metabolomics has emerged as a new “omics” platform in the last two decades with significant improvements in precision and sample throughput in the fields of analytical chemistry and mass spectrometry. Emergence of metabolomics has resulted in the creation of large datasets consisting of diverse classes of metabolites from multiple metabolic pathways. Metabolomics has been used to detect biomarkers of disease or drug-related changes between the control and experiment groups in a vast array of topics, such as Cancer [1]–[4], Diabetes [5], Cystic Fibrosis [6], [7], Toxicology [8]–[12], and Plant Research [13]–[17].

Univariate and multivariate statistical approaches have been used to analyze metabolites to determine meaningful biomarkers of disease presence/severity or treatment effectiveness. Univariate techniques include correlation/partial correlation analysis [15]–[19], ANOVA and significance testing for stand-alone metabolites [6]. These techniques consider one variable (metabolite) at a time. Multivariate techniques include Principle Component Analysis (PCA) [20], [21], Independent Component Analysis (ICA) [22], and Partial Least Squares–Discriminant Analysis (PLS-DA) [8], [11], [21], [23]. All of the multivariate analysis techniques noted above assume that the underlying dependencies among metabolites are linear, which is not necessarily the case. There are non-linear multivariate techniques in the literature like Non-Linear PCA [24]. However, we are not aware of their applications to metabolomics analysis, with the exception of Scholz et. al. [25], which tries to analyze time-course data with missing values.

It is important for domain scientists to see how each metabolite level changes with respect to a given condition (e.g., disease, treatment etc.), in order to hypothesize about the metabolic alterations in the variable group. Since multivariate techniques truncate variables (e.g., based on *variable importance in projection* scores in PLS-DA) to find a small number of components that explain the variance best, they are not a good fit for this use. Instead, researchers use univariate techniques to locate significant changes per metabolite between the variable and the control. Then, they map these changes onto a metabolic network in order to detect pathways with increased/decreased flux based on the significances of increases/decreases, and the number of metabolites that are significantly changed in a detected pathway [6]. This method causes a number of problems. First, the number of wild-type (control) and condition cohorts is usually small, and due to the high degrees of freedom, the test statistic may miss some changes as they do not show up as significant. Second, analyzing individual metabolites and aggregating the results may fail to explain the phenomenon at hand: it has been shown that different combinations of perturbed metabolites have different effects on the organism [26]. Third, when changes in two metabolites with respect to each other are analyzed, the significance of the change in the ratio of their concentrations is checked, which is an ad-hoc solution [27], [28].

Although current methods to analyze metabolite level changes are limited to univariate analysis, finding genes that are co-regulated with respect to a condition is a well-studied problem in the gene expression analysis context. Gene Set Enrichment Analysis (GSEA) is the first work that aims to find whether a predefined set of genes are enriched in a group of experiments with a condition [29]. GSEA has also been applied to metabolite data [30]. However, shortcomings of the method have been noted [31]. In another work, combinations of expression levels of genes are shown to be informative about a condition through mutual information (MI) [32], which is a statistical technique that can capture non-linear associations between random variables. In gene expression analysis, MI has been frequently used to find dependencies among gene expression profiles [33]–[36]. There are only a few mutual information-based techniques in the context of metabolomics analysis, targeting different problems such as reverse engineering of metabolic networks [37] or measuring correlations within the network [38].

**Figure 1:**
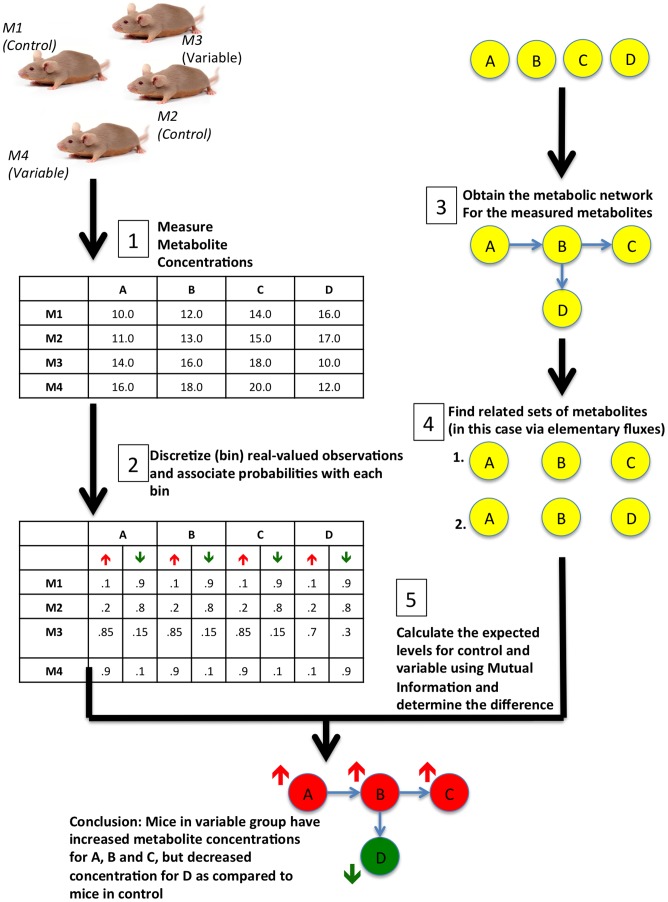
An overview for ADEMA. The first step is to construct a population such that it contains multiple individuals (in this case M1 and M2 who are in control group versus M3 and M4 who are in variable group. Concentrations of metabolites of interest are determined for all individuals (in this case concentrations of metabolites A, B, C and D). Then for the second step, each observation is assigned a probability to be in a discrete bin (we only consider two bins, namely, up or down). Third step is to construct the metabolic network to determine the associations between measured metabolites. In this figure circles represents metabolites and arrows represent the reactions that relate metabolites. This is followed by the fourth step that determines the subsets of metabolites, which are related in the metabolic network. We have found two sets, <A, B, C> and <A, B, D>, are the only subsets that are related. Using the probabilities found in step 2 and related subsets found in step 4, ADEMA determines control- and variable-specific metabolite levels (bins) and compares the changes in variable group with respect to mice in control group. In this example, ADEMA concludes that A, B and C are increased, and D is decreased in the variable group as compared to control mice.

In light of the limitations of the current approaches and motivated by the combinatorial approach used for gene expression analysis [32], we propose a novel multivariate method, called ADEMA (Algorithm for Determining Expected Metabolic Alterations). Given the control, ADEMA locates the “expected” metabolite changes that are indicative of the condition in the variable group. This task can help researchers (a) understand “under-the-hood” reasons for the symptoms that are being observed and (b) hypothesize on the cause-and-effect relationships between anomalies. Figure 1 provides an overview of the proposed methodology. The first step consists of forming a population with multiple individuals in variable and control groups and measuring the concentrations of those metabolites of interest. In the second step, each observation is assigned to discrete bins with some probability. The third step is to obtain the metabolic (sub)network for the measured metabolites. The fourth step locates the related subsets of metabolites using the metabolic network. The fifth and the final step uses the probabilities found in step 2, determines control-specific and variable-specific metabolite levels (bins), and compares them to find the changes in the variable group with respect to the control group. In the example of Figure 1, there are 2 mice in the control group and 2 mice in the variable group. Four metabolites of interest are measured for each individual and are related using the metabolic network. It has been determined that <A, B, C> and <A, B, D> are the related subsets. Each observation is assigned a probability of being either *up* or *down* (two discrete bins). Finally, the algorithm determines that mice in the variable group have higher levels of A, B, C, and decreased levels of D as compared to mice in the control group.

More specifically ADEMA has the following steps: (i) discretize (bin) metabolite observations using B-Spline curves, (ii) identify the related subsets of metabolites out of the observed metabolites by generating the Elementary Flux Modes (EFM) [39] of the metabolic network, (iii) locate combinations of metabolite pool levels (i.e., bins) that are “informative” with respect to a condition, and (iv) calculate the expected metabolite levels for the variable and the control groups, based on the marginal mutual information provided; and, compare them. By employing the identified expected levels, ADEMA can then be used as a classifier.

To evaluate ADEMA, a Cystic Fibrosis (CF) dataset (See Dataset S1) that consists of multiple 3-week-old wild-type (control) and CF (variable) mice is used. Although individual metabolite changes are not significant in 3-week-old mice, the expected levels found by ADEMA conform to the independently performed flux and gene expression analysis done on 3-week-old CF and WT mice. Moreover, we show that ADEMA can classify CF versus WT in three different datasets (See Dataset S1, Dataset S2, and Dataset S3). ADEMA can predict whether an unknown mouse has CF or not, with 1.0, 0.84, and 0.9 accuracy for each respective dataset. Results are better up to 31% as compared to other well-known classification algorithms.

## Methods

In this section, we describe how each subcomponent of ADEMA works. Please see Table 1 for the list of variables/terms and their explanations.

**Table 1:** List of variables/terms and their explanations.

Variables and Terms	Definitions
*M*	number of bins used for discretization of observations
*k*	number of bins an observation can be assigned to at the same time.
*n*	number of observed metabolites
*t*	knot vector that is used to define the shapes of B-spline curves
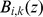	the probability associated with the *i* ^th^ B-spline curve for the given *z* value and for a specific *k*
*P*	population of individuals
*s*	an individual in the population
*m_j_*	*j* ^th^ observed metabolite
*s[m_j_]*	the observed value for *j* ^th^ metabolite for individual *s*
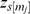	transformed value for s[*m_j_*] given the max and min values for m_j_
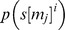	probability of assigning s[*m_j_*] to bin *i*.
*H(X)*	entropy of the random variable *X*.
*I(X;Y)*	mutual information of random variables *X and Y*.
*Sub*	a subset of the observed metabolites
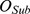	random variable that represents all combinations of the binned metabolite observations for *Sub*
*C*	random variable that represents the class variable (e.g. *WT* and *CF*)
*p_s_(o)*	probability of observing the bin combination *o* for individual *s*
*p(o)*	probability of observing the bin combination *o* in population *P*
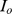	marginal mutual information obtained from the bin combination *o*
*o*[*m_j_*]	the bin associated with the *j* ^th^ metabolite in the bin combination array *o*
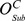	random variable that contains all bin combinations for metabolites in *Sub* that are class *C* -specific.
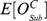	expected bins for metabolites in *Sub* for class *C*
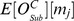	expected bin for metabolite *j* for class *C* found using the subset of metabolites *Sub_j_*
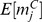	expected bin for metabolite *j* for class *C* found after aggregating results for different subsets

### Ethics Statement

All animal care and use was approved by the Institutional Animal Care and Use Committee of Case Western Reserve University.

### Binning Observations

Mutual information works with discrete values, whereas metabolite measurements are continuous real numbers. Therefore, to work with mutual information, one needs to discretize (bin) real values into discrete bins. In this subsection we discuss the existing methods employed in the literature and the reasoning behind picking a B-spline based strategy.

There are two types of methods in the literature to estimate probability densities out of continuous data: Parametric and Non-parametric methods [40]. The former one assumes that observations come from a known family of distributions. As we do not have any knowledge on the distributions of the observations we follow the latter approach (non-parametric).

**Figure 2:**
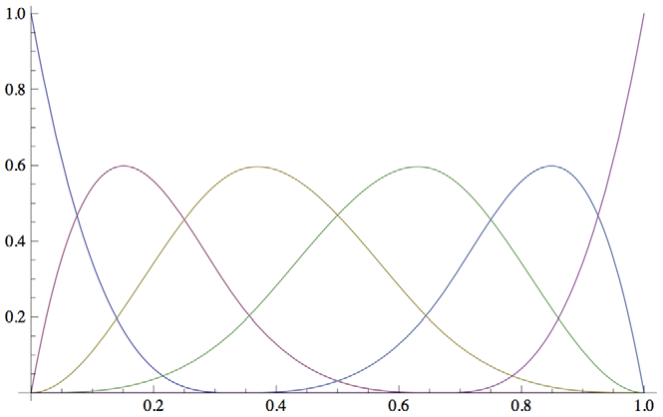
An example for B-spline basis functions. B-spline basis functions for 6 bins are shown. Each curve represents a bin. For each observation (*x-*axis), the corresponding *y* value on each curve yields the probability of that observation to be in that bin. Summation of the *y* values corresponding to an *x* value for all bins sum up to 1.

There are two non-parametric approaches in the current literature. The first one is kernel density estimation (KDE), which, given a window length *l*, estimates a density for each observation *x*, by counting the number of points in the window, weighted by their distances using a pre-selected kernel [39]. The result depends on the window length and the kernel used; also KDE has a high computational requirement. Thus, we did not pick KDE. The second approach is the histogram-based approach where observations are simply distributed into discrete bins. As metabolite measurements come with an error term, observations that are close to the borders can easily be misclassified when pre-determined thresholds are used [41], [42]. To address this issue, B-spline functions [43] have been used [44], [45]. Instead of placing an observation only in a single bin, each observation can be assigned to multiple bins, weighted by the B-spline function. In this case, bins are converted into overlapping polynomial functions. Figure 2 shows basis B-spline functions for 6 bins. In this figure each curve represents a bin. Each observation is assigned to the bin represented by the B-spline function (curve), with the corresponding probability for that observation. The sum of the probabilities for each bin is 1 for that observation. That is, for a specific *x* value in Figure 2, *y* values found using the B-spline curves would sum up to 1. In comparison, the histogram-based approach would divide the range [0, 1] in Figure 2, into 6 pieces (e.g., 0–0.16–0.33–0.5–0.66–0.83–1) and assign observations to only one of the bins (e.g., with probability 1 to the assigned bin).

In this paper, we make use of B-spline-based binning. The use of B-spline functions in our problem formulation requires two parameters, *M* and *k*. *M* denotes the number of bins. *k*, *k* ∈ [1, *M*], denotes the number of bins that an observation can be assigned to. Given *M* and *k*, the so-called “knot” vector *t* of length *M+k+1* is defined as follows:
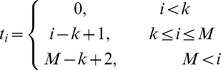
(1)This is called a uniform non-periodic knot vector [43]–[45]. After obtaining the knot vector, B-spline functions are defined recursively based on the knot vector as follows:
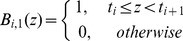
(2)

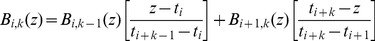
(3)Assume that we have a population *P*, and we have *n* observed metabolites, {*m_1_, m_2_, .., m_n_*}, for each individual *s* in *P*. Let *s*[*m_j_*] be the value of *j* ^th^ metabolite for individual *s*, where *j* ∈ [1, *n*]. Note that the domain of *z* in equations 2 and 3 is different from the domain (range of observations) for metabolite *m_j_*. Hence, we use the linear transformation defined in equation 4. *m_j_^min^ and m_j_^max^* are the minimum and maximum values observed in the population for *m_j_* respectively.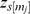 corresponds to the transformed value.
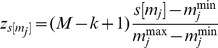
(4)The probability of 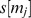 being assigned to bin *i* is denoted as 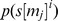, and is computed as in equation 5.
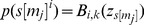
(5)Note that 
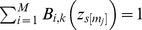
 That is, probabilities assigned to each bin for an observation sum up to 1. Then, for an individual *s*, the joint probability for any subset of metabolites to be in the given bins is found by multiplying probabilities of each metabolite in the subset to be in the corresponding bins. Once all metabolite measurements are associated with the corresponding bins, next step in the algorithm is to find related metabolites to be considered together.

### Selecting Subsets of Metabolites

ADEMA is a multivariate method that considers multiple metabolites at a time to capture interdependencies between molecules. There are two extremes. One can (i) calculate expected levels per metabolite, but then would miss the dependencies between metabolites themselves (e.g. consider all subsets of size 1), or (ii) look for expected states of all observed metabolites together (e.g. have only one subset that contains all metabolites), but, this time, would unnecessarily consider metabolites that are not related at the same time. Moreover, for *n* metabolites and *M* bins, there are *M^n^* possible combinations of metabolites and their corresponding levels (bins) as each metabolite can be in *M* different levels. As the method suffers from the curse of dimensionality, the subsets of metabolites to be considered together must be chosen carefully. Next we discuss three strategies to select related subsets of metabolites.

Metabolic networks provide a good understanding of the dependencies between metabolites by defining producer-consumer relationships. Elementary Mode Analysis [46] is a technique that identifies minimal sets of reactions that are active at the steady state of an organism and a metabolic network of interest. Each set is called an elementary flux mode (EFM), and any flux distribution on the metabolic network at steady state can be represented as a combination of the elementary modes. By definition, elementary modes define the subset of reactions that form the basis of the flux going through the metabolic network of interest. Thus, as a measure of dependency between metabolites, our first strategy for selecting related metabolite subsets is to use elementary modes, and consider all metabolites *associated with* the reactions in an elementary mode as a subset. In our context, association for a metabolite with a reaction means being a substrate or a product of that reaction. Note that elementary modes might still contain metabolites in the order of *O(n)*. Should this be the case, we break down EFMs into pieces using a predefined threshold that limits the maximum number of metabolites that can exist in a subset.

The second strategy for related metabolite subset selection aims to group metabolites that are close to each other in the metabolic network. For each metabolite, we construct a subset that contains all metabolites within one-hop distance to that metabolite (i.e., those that can be reached by a single reaction). The origin metabolite itself is also added to the set. Note that in the case of hub metabolites, the number of metabolites within a subset can still be large; thus, we apply the threshold strategy used for EFMs for this strategy as well. In contrast with the first approach, which locates the related metabolites using elementary fluxes that traverse the network, this approach disregards fluxes at the steady state, and focuses purely on topological closeness in order to determine the subsets.

The third strategy is to randomly pick distinct metabolites to form related subsets of metabolites. This strategy is used as a baseline strategy, assumes no prior metabolic network knowledge, and disregards all flux or topology based relationships among metabolites. The number of metabolites is again limited by a threshold. One advantage of the third strategy is that it can be used when there is no or limited knowledge about the metabolic network or when the network is very complex or large.

In the experimental evaluation, we compare performances of the three strategies in terms of the classification performance of ADEMA and report our findings on threshold selection and its effect on the algorithm efficiency. After the related subsets of metabolites are determined and the observations are discretized, the algorithm measures how informative the determined subsets are about the class variable (CF vs. WT) using mutual information.

### Determining Expected Metabolite Levels per Class

Mutual Information (MI) is an information theoretic technique to determine linear or non-linear statistical dependencies of variables. In our case, we would like to determine how much *CF* or *WT* genotype is reflected by discretized measurements (See *Binning Observations Subsection*) of subsets of metabolites (See *Selecting Related Metabolites Subsection*).

MI is based on Shannon Entropy, which measures the uncertainty associated with a random variable. Given a discrete random variable *X*, the entropy of *X* is denoted as *H(X)*. It is defined as in equation 6 where *p(x)* denotes the probability of observing *x* ∈ *X*.
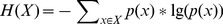
(6)Conditional entropy for *X* given *Y*, accounts for the uncertainty of *X* when *Y* is known, and is derived as in equation 7.
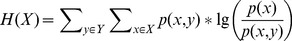
(7)Mutual Information *I(X;Y)* can be defined as the reduction in the uncertainty of a random variable when the other random variable is known (See equation 8). *I(X;Y)*, a real value in the range [0,1], is zero when observing one random variable does not give us any more information about the other.
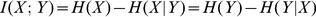
(8)In our context, for the subset *Sub* of observed metabolites, we are interested in the reduction of the uncertainty of the class variable *C*, given the binned versions of observations for *Sub*, namely 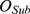. Equation 8 is equivalent to equation 9 below (the variables are renamed accordingly).
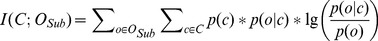
(9)There are *M* ^|*Sub*|^ possible combinations *in* 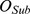. Note that the bin combination *o* in 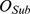 can be represented as an array of length |*Sub*| where each *o*[*m_j_*] ∈ [1, |*Sub*|] represents the bin for metabolite *m_j_* ∈ *Sub*. *p_s_(o)* is the probability of observing the bin combination *o* for individual *s*, and found in accordance with equation 5. Equation 10 shows the formula to find *p_s_(o)*. *p(o)* is the probability of observing *o* in population *P* and is found as shown in equation 11.
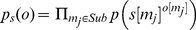
(10)

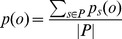
(11)Without loss of generality, we assume that *C* is a binary random variable, which can be either the control group or a variable group. Note that we take the liberty of using a binary random variable *C* for the sake of clarity, and our method can be generalized to beyond *C* being binary. As we compare wild-type mice with mice with Cystic Fibrosis disorder in the Results section, we name the control group as *WT* and a variable group as *CF*. Each combination *o* contributes to 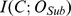 marginally, which is equal to the summation of information provided for *WT* and *CF* (see the outer summation in equation 9). We call this *marginal information* for *o*, and denote it as 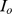, formally defined next.
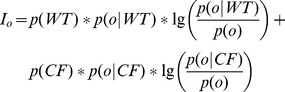
(12)Note that 
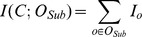
. In the CRANE algorithm of Chowdhury et al [32], each combination *o* is called a “substate”. CRANE searches for and uses the “informative substates” to train a neural network to classify samples in the gene expression analysis. Here, we have elected to classify the substates themselves based on the marginal information they provide for each class label. ADEMA uses all “substates,” instead of searching for the informative ones. Our approach (i) uses B-splines and (ii) attaches weights to each bin combination even when a combination has a low probability to occur. This enables ADEMA to use these substates for classification purposes instead of training a third party classifier. We exploit the following theorems.

#### Theorem 1



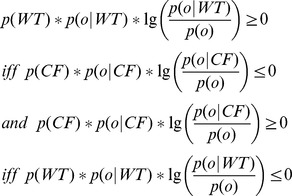



#### Proof for Theorem 1

Please see Text S1.

**Figure 3:**
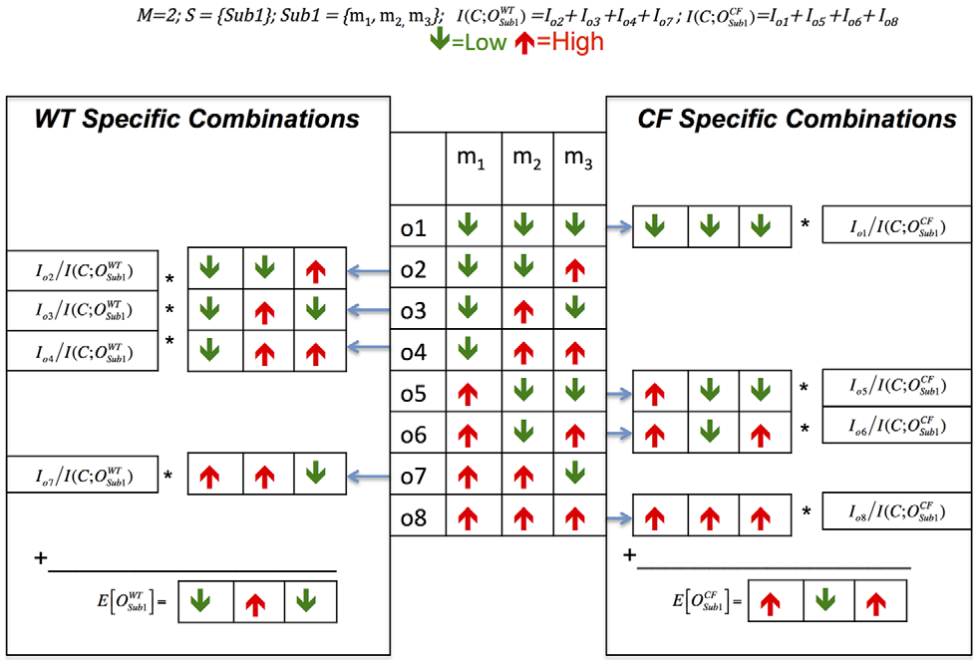
Illustration of determining *WT* and *CF* specific metabolite level combinations. Three metabolites are being analyzed to determine their expected levels for *WT* and *CF*. In this example, there is just one subset of metabolites considered, and there are two bins (e.g., either up or down). There are 2^3^ possible combinations of ups and downs. Using the function *ClassifyCombination*, it is determined that combinations o2, o3, o4, and o7 are *WT*-specific (on the left) and combinations o1, o5, o6, and o8 are *CF*-specific (on the right). When sets of combinations are weighed separately by their marginal information, expected levels for these metabolites for *CF* and *WT* are found.

Following Theorem 1, when one of the terms is positive (i.e., more frequently observed in that class), the other is forced to be less than that (i.e., it is less frequent in that class). As stated before, our goal is to locate the expected metabolite levels for *WT* and *CF*. We are seeking (i) the expected metabolic state occurs in *CF*, but not in *WT* and (ii) the expected metabolic state that is to occur in *WT* but not in *CF*. In order to do so, we classify each 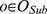 into one of the two following random variables: 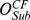 and 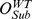 as indicators of *CF* and *WT*, respectively, based on 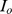. We make use of the following classification function:
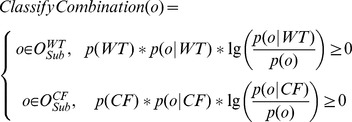
Note that 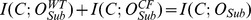. Then, we calculate the expected level (bin) for each metabolite in *Sub*. We find one expectation for *CF* and one for *WT* using sets 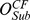 and 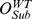 respectively. Intuitively, the associated probability for each combination 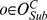 is defined to be 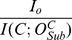 that reflects the marginal information provided by *o* among all other combinations that are informative about the class variable *C*. Note that, 
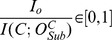
. Equation 13 defines the calculation of expectation. Simply, each index of *o* is multiplied by the associated probability, and the resulting sum is rounded to the nearest integer value.
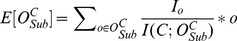
(13)
Figure 3 illustrates the essence of ADEMA with a simple example. In this illustration, there is just one subset of metabolites considered (e.g. a single EFM). There are three metabolites in the subset *Sub1*. We assume there are two bins (e.g. either up or down). Hence, there are 8 possible combinations of ups and downs for these 3 metabolites. In this hypothetical example, we determine that combinations o2, o3, o4, and o7 are *WT*-specific, and combinations o1, o5, o6, and o8 are *CF*-specific (using *ClassifyCombination* function). Then per group (CF vs. WT), each combination is weighed by the marginal information it provides and summed up to find the aggregation (using equation 13). Final metabolite levels found per group are considered as the representative expected levels for CF and WT for this combination of metabolites. Please also see Figure S3 for a toy example that shows the calculations.

**Figure 4:**
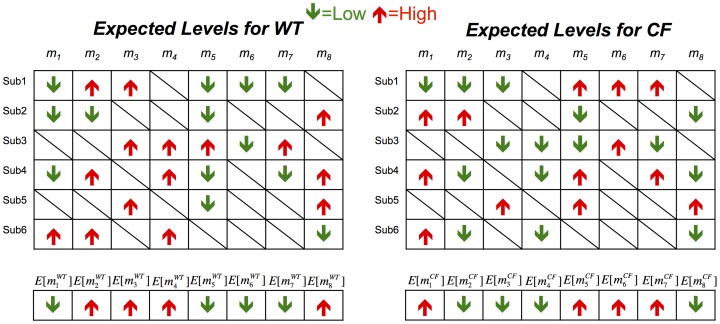
Illustration of combining expectations found by each EFM. In this illustration, there are 8 metabolites that are analyzed. We have 6 different subsets of metabolites found using EFMs. For each one of them, expected levels for *WT* (right) and *CF* (left) are found as explained in Figure 3. Individual expected levels are weighted using equation 14 to obtain a *WT-*specific and a *CF-*specific level for each metabolite.

As explained earlier, ADEMA may obtain more than one subset of metabolites using EFMs. After obtaining 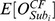 and 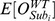
for each *Sub_i_*, ADEMA performs the following task of unifying results found per EFM. First, for each observed metabolite *m_j_*, it finds all *Sub*_i_ such that *m_j_* ∈ *Sub*_i_. We name this set of subsets of metabolites as 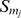. Then, ADEMA finds the expected level (bin) for each *m_j_* for each class as shown in equation 14 (denoted as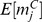). Real values are rounded to the nearest integers. The idea is to weigh the bin found by each EFM with the amount of MI it provides.
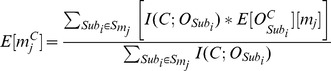
(14)
Figure 4 displays an example of this case. In Figure 4, there are 8 metabolites in the analyzed set and 6 subsets of metabolites are obtained using EFMs. After each subset is evaluated as depicted in Figure 4, their results are combined using equation 14 to obtain a *CF*-specific and a *WT*-specific level for each metabolite. As shown in the figure each metabolite subset contains a different combination of metabolites. For each metabolite, all subsets, which include that metabolite are determined. Then, each subset votes for the final prediction of the level of the metabolite. Predictions are weighed by the ratio of MI provided by the subset divided by MI provided by all subsets. Thus, the more informative the subset is, the more decisive its prediction is.

Finally, ADEMA finds the change in *CF* with respect to *WT* as the distance between 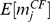 and 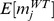. The sign of 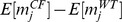 shows the direction of the change (increase, decrease or no change), and the magnitude shows the significance of the change.

To summarize, ADEMA first classifies each bin combination *o* as an indicator of either the variable or the control based on 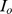. For each class, it determines the expected bin combination as a weighted sum of the classified combinations. They are weighted by the percentage of information they provide among all other combinations that are indicative of that class. This is done for each subset of metabolites considered. Finally, all expected levels found for each metabolite are combined as a weighted sum of the considered subsets; they are weighted by the percentage of mutual information each subset of metabolites provides among all the considered subsets. ADEMA finds an expected level per class for each metabolite. Next we describe an optional step of the algorithm. We show that the expected levels of metabolites can also be used to train ADEMA classifier to label unknown individuals.

### Classification Scheme for ADEMA

In this section, we show how ADEMA can be used as a classifier. The algorithm starts by generating the expected states for *CF* and *WT* as defined in equation 13 for the training data. The profile of individual *x* to be classified is binned using the same *M* and *k* values as the training sample.

After the binning is done, for each bin combination *o*, 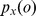 is found as shown in equation 10. Finally, *x* is classified using the following function, where *S* is the set of all subsets of metabolites considered:
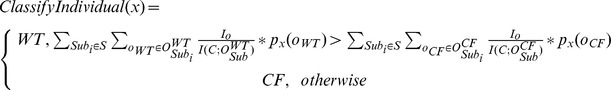
Essentially, the function computes the probability for *x* to be in the combinations that are indicative of *WT* and *CF*, weighted by the marginal information per combination, in a manner very similar to calculating the expected states as in equation 13. If it is more likely to be in “*WT*-indicative” states, then *x* is classified to be *WT*, and, otherwise, it is classified as *CF*.

### Datasets

We have used three *in vivo* datasets as analyzed in Bederman *et. al.* [47] and provided in the supplement. The first two datasets contain essential and non-essential fatty acid concentrations in the blood for two different aged mice cohorts: 3 weeks (Dataset S1) and 6 weeks (Dataset S2). We have obtained 13 metabolites for each of these datasets, namely, (i) essential fatty acids: *Linoleic Acid (C18:2ω6 (LA)), Arachidonic Acid (C20:4ω6 (AA)), Linolenic Acid (C18:3ω6 (ALA)), Eicosatetraenoic Acid (C20:4ω3 (ETA)), Eicosapentaenoic Acid (C20:5ω3 (EPA)) and Docosahexaenoic Acid (C22:6ω3 (DHA))*, and (ii) non-essential fatty acids: *Decanoic Acid (C10:0), Dodecanoic Acid (C12:0), Tetradecanoic acid (C14:0), Palmitic Acid (C16:0)*, *Palmitoleic Acid (C16:1), Stearic Acid(C18:0) and Oleic Acid (C18:1)*. There are 7 CF and 9 WT mice in Dataset S1 and 8 CF and 11 WT mice in Dataset S2. The third and final dataset contains the concentrations of 28 metabolites in the livers of another cohort of adult mice (Dataset S3). Those metabolites are *Alanine, Glycine, Valine, Leucine, Isoleucine, Proline, Urea, Serine, Threonine, Aspartate, Methionine, Glutamine, Oxo-proline, L-Phenylalanine, Tyrosine, Lactate, Glycerol, Succinate, Fumarate, β-alanine, Malate, PEP, alphaGP, Glucose, Citrate, Pantothenic acid, Uridine, and Inosine*. There are 12 WT and 10 CF mice in this dataset.

### Experimental Design

In this section we explain how we have applied ADEMA to the datasets described above. We implemented ADEMA in C# language and .NET Framework 4.0. All tests were performed on a Dell PowerEdge R710 Server with two Intel® Xeon® quad processors and 48 GB main memory, running the Windows Server 2008 operating system.

#### Binning observations

As described in the *Methods* section, the first step of the algorithm is to bin metabolite observations. Three datasets described above were input to the algorithm. For each observation, we obtained a probability per bin, i.e., the probability of that observation being in the specified bin. To choose the best set of parameters, we evaluated all combinations of *M*, *k* and *the maximum number of metabolites in a subset (maxSub)* such than 1≤*maxSub*≤7,2≤*k*≤3 *and* 3≤*M*≤6. We selected the following <*M,k,maxSub*> combinations per dataset as they provide the best accuracy: <6,3,8> for 3-week-old dataset, <3,3,7> for 6-week-old data set and <6,2,6> for the liver profile. Picking the best performing parameters with respect to the classification performance is also employed in the literature [32].

#### Selecting related metabolites

The next step in the algorithm is to select the related sets of metabolites. We employed all three strategies described in the *Methods* section.

To obtain the EFMs we used the YANA software package [48]. The networks were input using the visual interface of YANA. For the fatty acid data (Dataset S1 and Dataset S2), the metabolic network shown in Figure S1 was input as specified in Selway *et. al.* [49]. This network starts with Decanoic Acid, and produces Oleic Acid and Palmitoleic Acid. There are two other disconnected parts. The first path goes from Linolenic Acid to Docosahexaenoic Acid and the second path goes from Linoleic Acid to Arachidonic Acid. For the liver profile, we assembled the network by connecting the related metabolites in the dataset with reactions defined in the metabolic atlas by Selway [49]. The screenshot for Dataset S3 is shown in Figure S2. YANA produced 4 EFMs for the fatty acid datasets and 77 EFMs for the liver profile. The EFMs were broken into subsets when they had more than the number metabolites allowed per group (in this case, we fix this number to 8). There were 20 EFMs broken into two pieces for the liver profile, so we used 123 subsets of metabolites.

For the neighborhood approach, we obtained 1-neighborhood of each metabolite and constructed the metabolite subsets. For the fatty acid data, we obtained 11 subsets (all contain less than 8 metabolites) and, for the liver profile, we obtained 22 subsets of metabolites. Two of the subsets contained more than 8 metabolites; thus they were broken into two pieces to obtain 24 subsets in the end. Finally, to test the random strategy, we generated 4 random subsets for the fatty acid data and 123 subsets of metabolites for the liver profile each of which have less than 8 metabolites.

Table 2 shows the classification performances per metabolite selection strategy. EFMs achieve the highest accuracy in all cases. Therefore EFM based metabolite selection strategy is selected as our default metabolite selection strategy.

**Table 2:** Comparison of metabolite selection strategies.

	3-week data	6-week data	Liver profile
EFM	1	.78	0.81
1-Neighborhood	0.93	0.63	0.72
Random	0.68	0.68	0.81

Classification accuracies of each metabolite selection strategy per dataset are shown. For the random selection case, the number of subsets to consider is matched to the highest number of datasets among the competitors. Results show that the EFM strategy weakly dominates the competitors.

## Results

This section applies ADEMA to experimental metabolomics data on CF and wildtype mice, evaluates the results, and validates the approach. Cystic Fibrosis is an autosomal disorder caused by mutations in cystic fibrosis trans-membrane conductance regulator (CFTR), with the symptoms of respiratory and pancreatic dysfunction and low body-mass index. The most common mutation, F508del, results in deletion of a phenylalanine at 508^th^ amino acid position of the protein [50], [51].

### Determining Expected Metabolite Levels for 3-week-old CF Mice

In this section, we predict expected changes in the levels of metabolites for the 3-week-old *CF* and *WT* mice cohorts (See Dataset S1). We are using blood metabolite levels as surrogate markers for liver metabolism [52]. We obtain CF and WT specific metabolite level combinations and calculate the expectation per subset of metabolites. Finally, we aggregate the results for each subset found using EFMs. Please see Figure 1 for an overview of the method, and Figure S3 for an example.

Next, we test the validity of results generated by ADEMA against the findings of an independent wet-lab study. Details of the study are described in the next paragraph.

**Figure 5:**
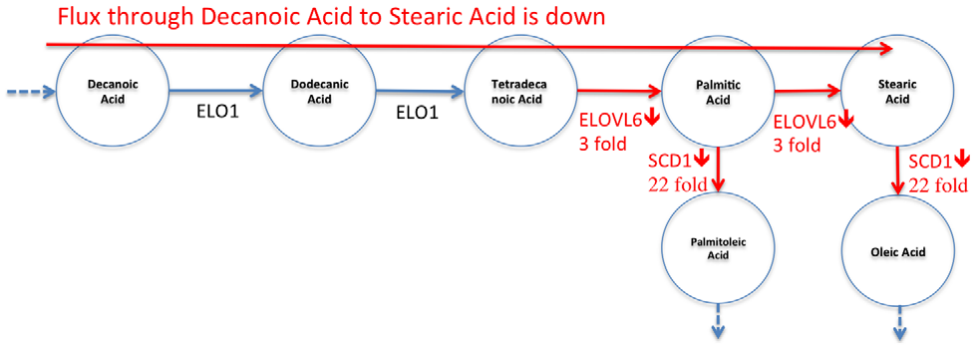
Results of gene expression analysis and flux measurements on DNL pathway. Circles represent the corresponding metabolites, and arrows represent reactions. *ELOVL6* and *SCD1* are the genes that express enzymes, which catalyze the corresponding reactions. This independent wet-lab study shows that (i) flux through Decanoic Acid to Stearic is decreased, and (ii) the shown genes that catalyze corresponding reactions are down-regulated in 3-week-old CF mice.

*Results* *of Independent Wet-lab Study on 3-week-old CF Mice* [*47*] *:* Using the incorporation of ^2^H from deuterated water administered to mice, (^2^H_2_O), it has been determined that *CF* mice had significantly lower *de novo lipogenesis* (*DNL*, conversion of carbohydrates to *Palmitic Acid*, and elongation to *Stearic Acid*). *DNL* was 75% lower in *CF* mice as compared to *WT*. This implies that the flux through the *DNL* pathway (Decanoic Acid - Stearic Acid) was drastically reduced. Figure 5 shows this change on the depiction of *DNL* pathway. It is not entirely clear why DNL rates were markedly decreased in *CF* mice; however, Bederman et al. found significantly decreased food intake in 3 week old mice (*CF* mice consume 50% less food) suggesting that carbohydrate/insulin activation of *DNL* pathway can be delayed in 3-week-old *CF* mice [47]. Consequently, *CF* mice have significantly decreased adipose tissue stores and delayed growth overall as adults. Also, gene expression analysis shows that the *ELOVL6* (elongation of Tetradecanoic to Palmitic Acid and subsequently to Stearic fatty acid) gene expression was down by 3-fold in CF mice. Similarly, the gene *SCD1* which expresses the enzyme that converts (desaturates) Palmitic Acid to Palmitoleic Acid and Stearic to Oleic Acid is down by 22-fold in CF mice. These changes are marked in Figure 5. Although gene expression levels do not have a one-to-one correspondence with reaction activities due to many factors such as post-transcriptional regulation, they have been used in the literature [53] as *cues* for reaction activity. Here, by considering the gene expression levels together with the reduction in *DNL* activity, it is safe to assume that the reactions are downregulated in the *CF* mice compared to *WT* mice.

**Figure 6:**
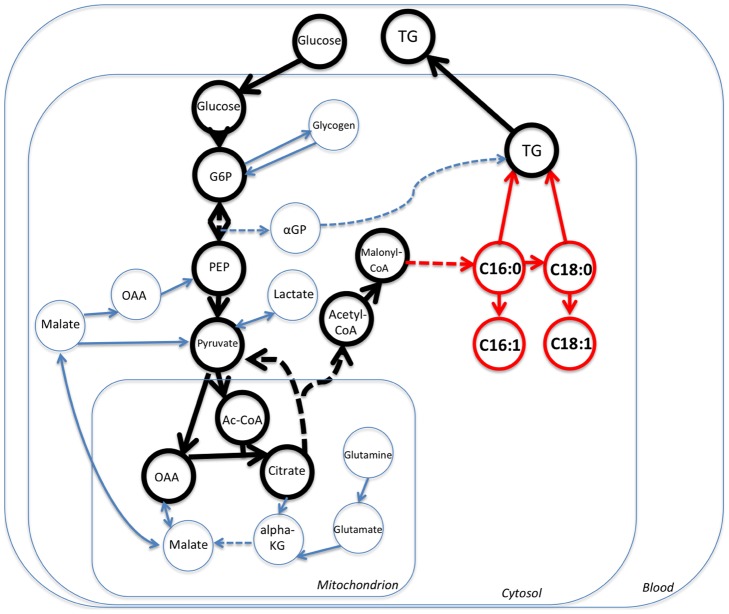
DNL pathway in the big picture. Circles represent the metabolites, and arrows represent reactions. Big rectangles represent compartments that reaction take place in (e.g., blood, cytosol, mitochondrion). DNL pathway holds an important place in the carbon flow of the liver cell. The glucose entering the cell can be utilized in the TCA cycle or can be converted to Triglycerides (TG) for storage. DNL pathway is particularly relevant to CF since it has been showed that mice with CF exhibit low lipogenesis and deposition of newly synthesize fatty acids into adipose tissue [47].

We show where the DNL pathway fits in the big picture in Figure 6. This figure shows general cellular metabolism with a focus on the lipogenic pathway. Bold arrows show carbon flux from Glucose into mitochondrion during the fed state. Since TCA cycle flux is slow in the fed state, excess carbon exits via citrate through citrate transporter back into the cytosol, where it is catalyzed by Citrate Lyase yielding Oxaloacetate (OAA) and lipogenic Acetyl-CoA, which is subsequently converted into Malonyl-CoA. Palmitic acid is then synthesized by adding units of Malonyl-CoA. Palmitate enters the DNL pathway, where it is elongated and/or desaturated to yield other components of the network that we describe in this manuscript. This overall DNL pathway is particularly relevant to CF due to the facts described above. Thus, examining carbon flux through this lipogenic network allows us to answer clinically relevant questions in CF research.

**Figure 7:**
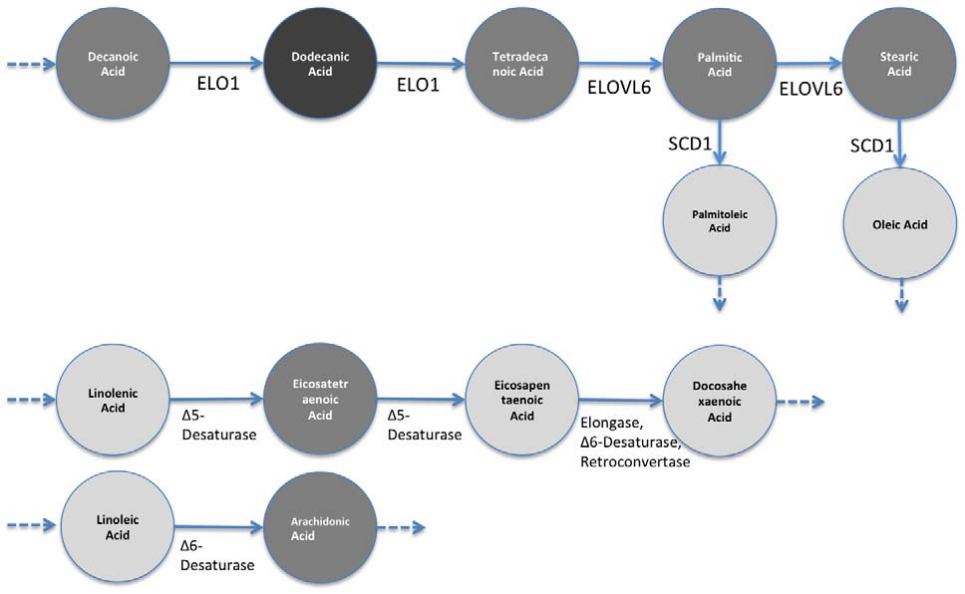
Results of significance testing for individual metabolites on DNL Pathway. Dark grey-colored metabolite represents significant increase for a metabolite in *CF*, compared to *WT* (3-week-old mice). Grey represents “no significant change”, dark grey represents “significant increase”, and light grey represents “significant decrease”. Significance tests are done using student's *t* test per each metabolite independently. The results show that the path Decanoic Acid to Stearic shows no significant change other than an increase in Dodecanoic Acid even though (1) the flux is shown to be decreased on this path, and (2) *ELOVL6* expression level is lower.

Figure 7 shows the results when only individual metabolites are tested for significant changes using the Student's t-test. That is, one by one, each metabolite is tested to see whether the change is significant or not. The result shows that there is no significant change (marked with grey) in the *DNL* pathway (Decanoic Acid to Stearic Acid) other than an increase for Dodecanoic Acid (marked with dark grey). These conclusions do not comply with the data or with the gene-expression-level-based expectation noted above. One would expect a drastic change on the metabolite values as the evidence suggests that there is a substantial alteration on the pathway flow. The only point, which is in line with the independent study, is that there are decreases of Palmitoleic Acid and Oleic Acid levels which agree with drastically low SCD1 levels.

**Figure 8:**
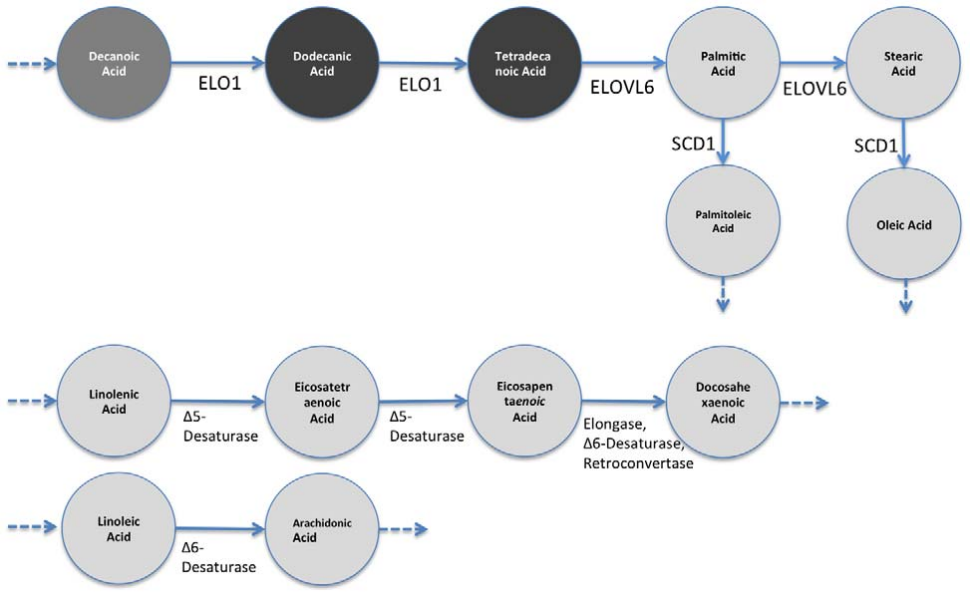
Expected level changes found using ADEMA for metabolites on DNL Pathway. Coloring scheme is the same as in Figure 7. Resulting expected metabolite changes are computed using ADEMA, for the *CF* mice w.r.t. *WT* mice (3-week-old mice). We see that Palmitic Acid and Stearic are decreased, as suggested by the flux measurement and *ELOVL6* levels. The increases in Dodecanoic Acid and Tetradecanoic Acid can be explained by a downstream effect of Stearic and Palmitic Acid that lead to the accumulation of these two metabolites as they are no longer consumed.

Figure 8 shows expected metabolite level changes for *CF* mice with respect to *WT* mice found by ADEMA. We set *M = 6*, *k = 3 maxSubset = 8*, which provide the best classification performance as shown in *Classification Performance Section* below. Unlike the results in Figure 7, we find that Palmitic Acid and Stearic are expected to decrease in a 3 week-old *CF* mouse, which supports the independent results. ADEMA's prediction shows that Dodecanoic Acid and Tetradecanoic Acid are increased. The increases in Dodecanoic Acid and Tetradecanoic Acid can be explained by a downstream effect of Stearic Acid and Palmitic Acid that lead to accumulation of these two metabolites as they are no longer consumed (Note that Palmitic Acid and Stearic Acid have bigger pool sizes than the precursors). Finally, ADEMA predicts that all metabolites in essential fatty acid elongation pathways Linolenic Acid to Docosahexaenoic Acid and Linoleic Acid to Arachidonic Acid are decreased. When metabolites are analyzed one by one, one would argue that there are no significant changes, which would lead to a different conclusion than the independent study. ADEMA provides a more consistent scenario, where the main products of the pathway are all decreased and lead to the accumulation of the precursors.

### Classification Performance

To present the classification performance of ADEMA (See Methods), we make use of the blood profiles at 3 and 6 weeks (3 and 6 weeks data mentioned above) and the liver profile. To test the ADEMA approach, leave-one-out cross validation (LOOCV) is used. That is, we remove a mouse from the dataset (test data), train the classifier using the rest of the dataset (training data) and blindly classify the removed mouse. We repeat this for each mouse in that dataset. Note that LOOCV is desirable for small data size, is almost unbiased and is frequently used in microarray studies, despite the high computation model building cost [54]. We report accuracy, precision and recall results along with the F-measure. F-measure is the harmonic mean of precision and recall.

**Figure 9:**
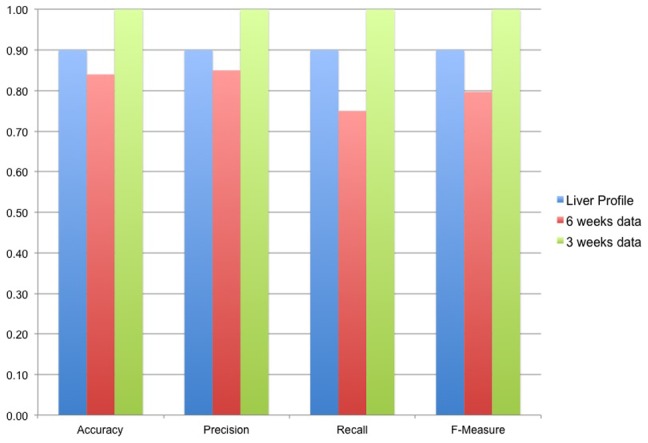
Classification performance for ADEMA on 3 *in vivo* datasets. Accuracy, Precision, Recall and F-measure results are shown for datasets S1, S2, and S3. The accuracy of the classifier is significant for all datasets (two-tailed Fisher's exact test).

Results for the classification tests are shown in Figure 9. ADEMA was able to predict if an unknown individual is *CF* or *WT* with an accuracy of 1 in Dataset S1, 0.84 in Dataset S2 and 0.9 in Dataset S3. Applying Fisher's exact test (two-tailed) to the results we find that our classifiers have *p*-values of 3*10^−4^, 6.3*10^−3^ and 1.323*10^−5^ for Dataset S3, Dataset S2 and Dataset S1 respectively. Hence, the accuracy of the method is statistically significant in all datasets. Note that to perform classification of the 3-weeks data, ADEMA uses the *CF-* and *WT-* specific metabolite levels, which are also used to obtain Figure 8.

**Figure 10:**
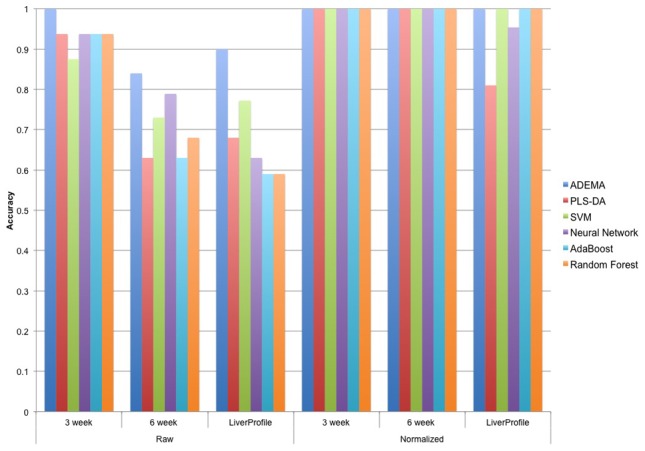
Comparison of ADEMA with other classifiers. Figure shows the comparison of ADEMA's accuracy with other well-known non-linear classifiers. For PLS-DA, MetaboAnalyst's implementation is used, and for the rest of the techniques, WEKA implementations with default parameters are used. We report classification results for raw data and data that is normalized using the method described by Dubitzky et al [54]. Results show that ADEMA performs up to 31% better than the other methods, and performs better than all other methods in at least one dataset.

Next, we compare the accuracy of our classifier with other non-linear classifiers from the literature: PLS-DA, Random Forest, SVM, AdaBoost, and Neural Network. For PLS-DA, MetaboAnalyst's implementation is used [30]. For the rest of the methods, WEKA implementations [55] (SMO, RandomForest, AdaBoostM1, MultiLayerPerceptron respectively) are used with default parameters. Results for classification using normalized and raw data are shown in Figure 10. The normalization technique presented by Brodsky *et al.* [56] is used. This is a normalization technique tailored for metabolomics analysis, with the goal of minimizing errors committed on the peak picking and alignment procedures done on LC-MS based metabolomics data. This method first performs quantile normalization on each intra-replicate group, then performs a quality control to adjust its parameters to minimize inter-replicate discrepancies. Application of these methods to the datasets is straightforward. The dataset itself (real values), or the normalized version, was input to the method, and the classification accuracy is returned.

From Figure 10, for all normalized sets and raw 3-week data, all classifiers return statistically significant results at 0.05 level. However, for the 6-week data only ADEMA and Neural Network, and for the liver profile only, ADEMA and SVM return statistically significant accuracies. Results show that ADEMA performs equivalent or more accurately in all cases (up to 31%), and it performs better than all other methods for at least one dataset. Results also show that normalizing the data results in better accuracy for all approaches, with improvements up to 42%. Although in some cases performances of ADEMA and the other methods are identical, the advantage comes from the interpretability of ADEMA's result. That is, all the other algorithms make a prediction using some internal techniques, but provide no feedback or biological explanation to the user about how they did it or what made them to predict what they predicted. For instance, PLS-DA uses the most significant variables (in our case metabolites) that explain the variance, and disregards the rest of the variables, which makes it impossible to evaluate all metabolites at hand. SVM is known for its lack of interpretability as it transforms the variable into a high dimensional space to perform classification. Neural networks use a layered network structure where each node assigns weights to the interconnections; and, the output is a binary classification decision. The Random-Forest method builds multiple classification trees, and performs a majority voting among them. Although individual trees are interpretable (e.g., that, say, A is low and B is high implies *CF*), the majority voting obscures the interpretability of the final result. Finally, AdaBoost tries to improve the performance of the underlying classifier by reassigning weights to the misclassified examples in the previous iterations. AdaBoost is an optimization algorithm that relies on another classification algorithm, and the interpretability of the result depends on the underlying algorithm; in the end, the output is a binary decision. In comparison, ADEMA outputs expected levels, and outputs a snapshot of the metabolic changes that have led to the classification conclusion. This way, ADEMA lets researchers to hypothesize on the metabolic activity that distinguishes variable from the control. Once again, classification scheme for ADEMA uses the same *WT-* or *CF*-specific combinations that have been found to predict the expected levels as shown in Figure 8. That is, during classification, it uses these combinations to calculate whether it is more likely for the unknown individual to be in the CF-specific states to WT-specific states. Therefore, Figure 8 provides an interpretation of the classification decisions ADEMA has made. Moreover, from the results of all classification tests, we conclude that ADEMA provides biologically meaningful signatures to predict the expected levels that can also be employed for classifications of samples.

### An In-Silico Experiment to Validate Expected Levels

To further validate the expected levels found by ADEMA, we generated an *in silico* dataset using the kinetic model of simplified Glycolysis (Dataset S4). We used the Wolf2000_Glycolytic_Oscillations model [57] from BioModels Database [58]. Using the online simulation interface of the PathCase^SB^ system [59], [60], we ran 10 independent simulations using different initial concentrations for Glucose, which is the only incoming source of flux in the network (boundary metabolite). We ran 5 simulations with initial Glucose concentrations smaller than 6 units, and considered them as the control group. Then, we ran 5 simulations with initial Glucose concentration larger than 10 units and considered this group the variable group. Per each simulation, we obtained 75 values per metabolite for 75 time points and averaged them into a single representative amount. In this dataset, we obtained concentrations for 9 metabolites, which are reported in the model. These metabolites are: *Glucose*, *Fructose 1,6 Bisphosphate, Glyceraldehyde 3-Phosphate+DHAP (abstracted as a single metabolite in the model), 3 Phosphoglycerate, Pyruvate, Acetaldehyde*, and *External Acetaldehyde*.

Observations are discretized using B-splines as described before. We picked <6,2,8> as the <*M,k,maxSubset*> combination based on the classification performance. YANA returned a single EFM (with all 9 metabolites), which is then broken into two subsets.

**Figure 11:**
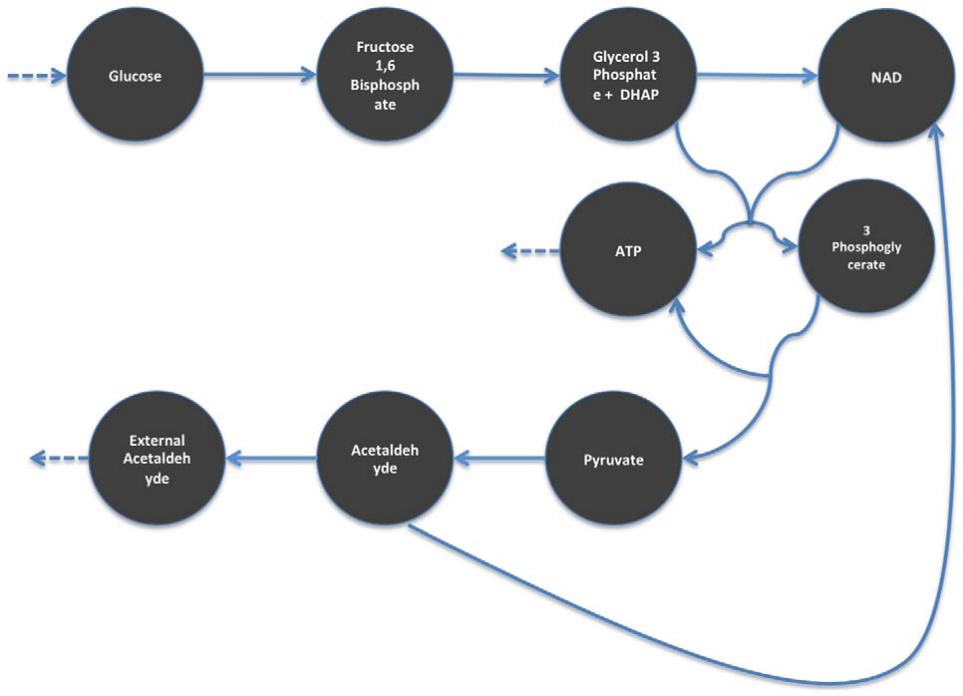
Predicted Metabolite Levels for the In Silico Dataset. This figure depicts the simplified Glycolysis pathway as described by the BioModels model Wolf2000_Glycolytic_Oscillations. Figure shares the legend of Figure 7. As the variable group has increased Glucose levels, and, therefore, increased input to the model, the expectation is to observe an increase in the overall metabolite levels. As expected ADEMA predicts that every single metabolite is increased in the variable group, with respect to the control group.

As the initial input to the metabolic network was increased (i.e., increased Glucose concentrations) in the variable group, the expectation is to observe an increase in the metabolic activity along the network and increased metabolite concentrations. However, student's t-test cannot detect any significant changes between two groups for ATP and *Fructose 1,6 Bisphosphate* levels. On the other hand, ADEMA predicted an increase for all metabolites in the variable group with respect to the control group. Results are shown in Figure 11. This also supports the reliability of the expected levels found by ADEMA.

### Time Performance

**Figure 12:**
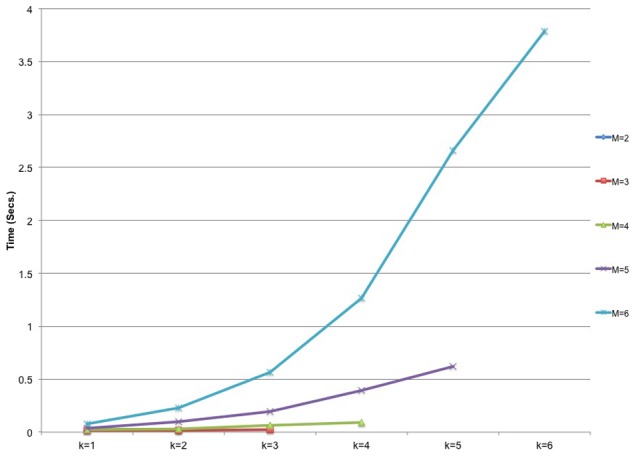
Time Performance of ADEMA on Dataset S1. Time requirements for changing *M* and *k* values show exponential increase for 3-week-old data.

For the picked parameters described at the beginning of this section, ADEMA took 17 seconds for Dataset S1, 0.05 seconds for Dataset S2, and 66 seconds for Dataset S3 to classify an unknown individual on average. ADEMA requires more time as each of *M*, *k*, the number of subsets, and the subset size increase. Parameter *k* increases the time requirement because of the recursive computations shown in equation 3. As discussed in Methods, we limit the maximum size of the subsets of metabolites; so, we show the effect of the rest of the variables noted above in Figure 12.

In Figure 12, we show the average time required to calculate the expected levels per metabolite and to classify a mouse, for given *M* and *k* values using Dataset S1. Figure 12 clearly shows that, as parameters *M* and *k* increase, the computation time increases exponentially. Although this may raise a question on the applicability of ADEMA in a more general setting with networks of larger sizes and increased number of EFMs, next we show that ADEMA's parameters can be relaxed to trade accuracy for time. Table 3 shows accuracy results for all <*M,k,maxSubset*> combinations tested on Dataset S3. Table 4 lists the time taken for each respective test. As indicated before, the best accuracy result (0.9) was obtained using the combination <*6,2,6*>. This particular test took ∼66 seconds as shown in Table 4. On the other hand, the combination <*4,2,5*> resulted in 0.77 accuracy which is also significant at the 0.05 level, and took only ∼2 seconds. Thus, the execution time of the algorithm can be limited by relaxing the parameters, while still providing statistically significant classification performance. The accuracy/time performance tables for Dataset S1 are shown in Table S1 and S2, respectively, and for Dataset S2 they are shown in Table S3 and S4, respectively. Similarly, when the metabolic network is large and the EFM calculation takes a long time, the algorithm can be switched to using the random metabolite selection strategy. The modular structure of the algorithm enables the user to pick parameters, or to switch between the subcomponents of the algorithm to achieve accuracy within the time limits set by the user for larger problems.

**Table 3:** Accuracy of ADEMA Classification Scheme on Dataset S3 w.r.t. Varying Parameters.

		M = 3		M = 4		M = 5		M = 6	
		k = 2	k = 3	k = 2	k = 3	k = 2	k = 3	k = 2	k = 3
Max Subset Size	2	0.5	0.5	0.63	0.59	0.5	0.63	0.59	0.68
	3	0.59	0.54	0.72	0.63	0.72	0.72	0.68	0.72
	4	0.59	0.59	0.72	0.72	0.68	0.77	0.72	0.77
	5	0.68	0.59	0.77	0.72	0.72	0.77	0.86	0.77
	6	0.59	0.54	0.72	0.77	0.81	0.72	0.90	0.72
	7	0.5	0.54	0.72	0.72	0.81	0.77	0.86	0.77

Figure shows how accuracy of ADEMA classifier changes with respect to changing parameters <*M, k, maximum subset size*>. The best result is obtained for the combination <6,2,6>.

**Table 4:** Execution time of ADEMA Classification Scheme on Dataset S3 w.r.t. Varying Parameters.

		M = 3		M = 4		M = 5		M = 6	
		k = 2	k = 3	k = 2	k = 3	k = 2	k = 3	k = 2	k = 3
Max Subset Size	2	0.24	0.25	0.19	0.2	0.37	0.25	0.47	0.45
	3	0.27	0.19	0.33	0.34	0.66	0.52	0.7	0.94
	4	0.24	0.35	0.53	0.68	1.16	1.70	2.36	4
	5	0.39	0.47	1.23	2.01	3.45	7.68	8.72	25.16
	6	0.96	1.19	4.52	12.2	20.5	80.8	65.8	334
	7	1.64	2.77	11.5	37.7	61.3	276.5	217.8	1178

Figure shows how much time (in seconds) it takes for the ADEMA classifier to train and classify an unknown individual on average for different parameter combinations <*M, k, maximum subset size*>.

To further validate that ADEMA can be applied on large-scale networks we have tested the algorithm on two in silico datasets generated for models Bungay2003_ Thrombin_ Generation [61] and Ung2008_EGFR_Endocytosis [62]. Former model has 74 species (metabolites) and latter model has 194 species. We have generated the data following the same procedure to generate data for Wolf2000_Glycolytic_Oscillations model as described in the previous subsection. For the first model we have run 5 simulations with low initial concentrations for “Ps_f” (<1500), which represent the *WT* group and 5 simulations with high concentrations for “Ps_f” (>2800) which represent the *CF* group. Same is done for “Src” in the second model (low concentrations <6 and high concentrations >20). Again, per simulation, we obtained 75 values per metabolite for 75 time points and averaged them into a single representative amount. For both datasets we tested ADEMA's classification scheme using LOOCV. ADEMA was able to achieve perfect accuracy for both datasets, and took only 0.96 and 0.86 seconds on average, respectively. Results show that ADEMA can be applied on large networks without sacrificing accuracy or reliability.

## Discussion

ADEMA is a new framework that identifies expected level changes for metabolites with respect to a condition. For each related set of metabolites, it calculates the mutual information between each combination of discretized levels of the metabolites in that set, and the class variable. We have shown how each combination can be classified as being informative in terms of the variable group or the control group, and have used this information to calculate the expected levels per class variable. ADEMA also presents a scheme to use expected levels to classify individuals with unknown class labels. We have shown that the expected metabolite level changes calculated by ADEMA conform to flux measurement results and the gene expression analysis done on 3-week-old CF mice. We have also shown that ADEMA's classification performs more accurately than five other well-known classification techniques by up to 31%. Unlike all other classification techniques, ADEMA's classification results are also interpretable. That is, ADEMA provides an explanation of the classification result by outputting the expected level changes, along with the prediction. We think that this feature is very important for metabolomics researchers who attempt to capture a snapshot of the metabolism, and understand the differences between the two groups.

ADEMA attempts to minimize the loss of biological information contained in a metabolic profile. Preservation of information is particularly important when a disease causes subtle changes in metabolite levels, i.e., changes that are insignificant at a single metabolite level, but significant when taken together with other metabolite levels.

In terms of Cystic Fibrosis, our hope is for ADEMA to contribute to the biomarker potential of dyslipidemia in Cystic Fibrosis. Fatty acid profiles are currently used as outcome measures in clinical trials for CF patients; the use of ADEMA would maximize the amount of information obtained from fatty acid profiles, improving the outcome measure sensitivity. Metabolite profiles are useful in the treatment of other diseases as well. For instance, comprehensive serum fatty acid profiles are used to diagnose and monitor individuals with inborn errors of mitochondrial fatty acid oxidation and peroxisomal disorders [63]. ADEMA's increased sensitivity to subtle changes in metabolite levels may be beneficial to the analysis of metabolite profiles in many diseases. Furthermore, the advent of a new class of CFTR potentiator drugs (i.e., VX-770, discussed in Ramsey et al. 2011 [64]) obviates the need for additional outcome measures in drug trials. Fatty acid levels were not reported as an outcome measure in Ramsey et al. 2011, perhaps because of unresolved inconsistencies in the direction of change in individual fatty acids [65]. Further research is needed to determine if analysis of fatty acid profiles by ADEMA will provide a more clinically useful outcome measure.

We foresee that there is room for improvement in ADEMA on selecting the relevant subsets of metabolites. Rather than relying on the existing knowledge of relations between metabolites, one can search for signatures [32] that define the dataset to reach higher levels of mutual information. This may benefit the calculation of expected levels of metabolites and classification. Another limitation with ADEMA is its exponential nature (See Results). However, as described in the algorithm can be tweaked to trade accuracy for execution time. Searching for small, but informative, states may also reduce the time complexity of ADEMA.

ADEMA fills an important gap in the metabolomics literature because it provides an analysis of non-linear dependencies among multiple metabolites, and derives an expectation of changes with respect to a condition. This is a question that all “omics” platforms seek an answer for, and the need for techniques that embrace transcriptomics, proteomics and metabolomics data is substantial. ADEMA has no metabolite-specific dependencies other than the use of EFMs, and it can easily be incorporated to other high-throughput techniques.

## Supporting Information

Dataset S1 **Metabolite measurements for 3-week-old mice.** This data is referred as 3 week data in the text and contains blood measurements for metabolites of DNL pathway.
(DOC)

Dataset S2 **Metabolite measurements for 6-week-old mice.** This data is referred as 6 week data in the text and contains blood measurements for metabolites of DNL pathway.
(DOC)

Dataset S3 **Liver profile for adult mice.** This data is referred as liver profile in the text and contains blood measurements for metabolites of DNL pathway.
(DOC)

Dataset S4 **In Silico Dataset generated using Wolf2000_Glycolytic_Oscillations Model.** We have generated the following data by running 10 distinct simulations on Wolf2000_Glycolytic_Oscillations using different initial concentrations for Glucose. For each metabolite in each experiment we have obtained 75 values (there were 75 time points) and averaged them to obtain a representative value. We assumed variable group had higher (>10) initial Glucose concentrations and control group had low (<6) Glucose concentrations.
(DOCX)

Figure S1 **YANA screenshot of the network created to obtain EFMs for Dataset S1 and Dataset S2.** In this figure blue circles represent internal metabolites and pink circles represent external metabolites. External metabolites are not considered in the analysis, but they are input to specify the entrance and exit points to the network. Rectangles represent reactions that relate metabolites. These reactions are “abstract” reactions that might contain one or more reactions. This network represents the DNL pathway and was used to obtain the EFMs.
(DOC)

Figure S2 **YANA screenshot of the network created to obtain EFMs for Dataset S3.** Colors and shapes representing entities are same as in Figure S1. This network is formed by linking related metabolites together according to Selway et al. [51] and was used to obtain EFMs.
(DOC)

Figure S3 **Example that shows basic calculations done for ADEMA.** Given one individual per class and two measured metabolites, ADEMA generates 4 possible metabolite combinations and based on the probabilities obtained using B-spline curves (in this case estimates) expected levels per group are found. ADEMA first classifies bin combinations as WT- and CF-specific to conclude that ↑↑ are the expected levels for CF and ↓↓ are the expected levels for WT.
(DOC)

Table S1 **Accuracy results for different** ***M,k and max subset size*** **parameters for Dataset S1.**
(DOC)

Table S2 **Accuracy results for different** ***M,k and max subset size*** **parameters for Dataset S2. Best result is marked as bold.**
(DOC)

Table S3 **Time results (secs) for different** ***M,k and max subset size*** **parameters for Dataset S2.**
(DOC)

Table S4 **Time results (secs) for different** ***M,k and max subset size*** **parameters for Dataset S2.**
(DOC)

Text S1 **Proof for Theorem 1.**
(DOC)
