# Supplementary material for: ADEMA: An Algorithm to Determine Expected Metabolite Level Alterations Using Mutual Information
Source: PLoS Comput Biol. 2013 Jan 17;9(1):e1002859. doi: 10.1371/journal.pcbi.1002859 (PMC3547803; doi:10.1371/journal.pcbi.1002859)
Supplement: Dataset S1 — Metabolite measurements for 3-week-old mice. This data is referred as 3 week data in the text and contains blood measurements for metabolites of DNL pathway. (DOC) [file pcbi.1002859.s001.doc]

**Dataset S1. Metabolite measurements for 3-week-old mice.** This data is refered as *3 week data* in the text and contains blood measurements for metabolites of DNL pathway.

| ID | Genotype | Sex | C10:0 | C12:0 | C14:0 | C16:1 | C16:0 | C18:1 | C18:0 | C18:2 (LA) | C18:3 (ALA) | C20:4 (AA) | C20:3 | C20:5 (EPA) | C22:6 (DHA) |
| --- | --- | --- | --- | --- | --- | --- | --- | --- | --- | --- | --- | --- | --- | --- | --- |
| 8864 | WT | F | 0.29360 | 0.85904 | 2.20407 | 0.33345 | 75.34592 | 7.27162 | 81.15719 | 6.69135 | 0.13145 | 0.07062 | 0.07048 | 0.00885 | 0.00743 |
| 8865 | WT | F | 0.12985 | 0.52823 | 1.29348 | 0.18023 | 32.98228 | 3.47492 | 33.16531 | 2.92297 | 0.05714 | 0.04427 | 0.04477 | 0.00536 | 0.00494 |
| 8874 | WT | F | 0.14516 | 0.44398 | 1.31298 | 0.23537 | 35.76914 | 4.88123 | 37.72331 | 4.62872 | 0.09328 | 0.08815 | 0.08709 | 0.01122 | 0.01191 |
| 8875 | WT | F | 0.11387 | 0.34175 | 0.87919 | 0.14344 | 24.52840 | 2.57382 | 27.61215 | 2.33519 | 0.04748 | 0.03571 | 0.03566 | 0.00426 | 0.00349 |
| 8881 | WT | M | 0.10346 | 0.52035 | 1.30206 | 0.21523 | 26.31079 | 3.82472 | 26.62785 | 3.30301 | 0.06605 | 0.04848 | 0.04809 | 0.00546 | 0.00678 |
| 8882 | WT | M | 0.11778 | 0.48814 | 1.50104 | 0.23623 | 27.05596 | 3.90297 | 27.34638 | 3.24551 | 0.07179 | 0.04316 | 0.04326 | 0.00510 | 0.00592 |
| 8885 | WT | M | 0.10443 | 0.23093 | 0.81813 | 0.14973 | 25.02495 | 3.35340 | 26.88659 | 3.30397 | 0.07207 | 0.03234 | 0.03234 | 0.00636 | 0.00630 |
| 8889 | WT | M | 0.12769 | 0.43106 | 0.96098 | 0.12810 | 24.12896 | 2.50828 | 26.97738 | 2.29916 | 0.04195 | 0.04155 | 0.04156 | 0.00276 | 0.00408 |
| 8892 | WT | M | 0.10990 | 0.25801 | 0.77296 | 0.15215 | 23.01875 | 2.75134 | 25.19257 | 2.49193 | 0.04645 | 0.04301 | 0.04307 | 0.00430 | 0.00528 |
| 8915 | CF | F | 0.11475 | 0.73150 | 1.28170 | 0.11916 | 22.89073 | 1.88024 | 25.23165 | 1.81930 | 0.02448 | 0.04648 | 0.04639 | 0.00236 | 0.00377 |
| 8916 | CF | F | 0.11477 | 1.10509 | 1.98201 | 0.11045 | 24.35132 | 2.21807 | 26.51612 | 2.10598 | 0.02921 | 0.05330 | 0.05329 | 0.00227 | 0.00348 |
| 8962 | CF | F | 0.09590 | 0.78734 | 1.26701 | 0.08450 | 23.68183 | 1.64115 | 27.61670 | 1.38167 | 0.02102 | 0.02153 | 0.02150 | 0.00061 | 0.00149 |
| 8862 | CF | M | 0.10582 | 0.38263 | 0.76532 | 0.08452 | 21.41601 | 1.71063 | 24.83843 | 1.46160 | 0.02126 | 0.04254 | 0.04261 | 0.00169 | 0.00294 |
| 8863 | CF | M | 0.13887 | 0.40975 | 0.83231 | 0.09442 | 23.45811 | 2.03318 | 26.64722 | 1.79510 | 0.02937 | 0.06236 | 0.06222 | 0.00188 | 0.00371 |
| 8884 | CF | M | 0.10256 | 0.53676 | 1.08569 | 0.09950 | 22.68349 | 2.11981 | 24.98502 | 1.59392 | 0.02197 | 0.04628 | 0.04625 | 0.00174 | 0.00375 |
| 8965 | CF | M | 0.11908 | 1.05721 | 1.78900 | 0.11311 | 24.15923 | 2.30210 | 26.10228 | 1.80471 | 0.02960 | 0.04610 | 0.04608 | 0.00139 | 0.00334 |
