# Supplementary material for: ADEMA: An Algorithm to Determine Expected Metabolite Level Alterations Using Mutual Information
Source: PLoS Comput Biol. 2013 Jan 17;9(1):e1002859. doi: 10.1371/journal.pcbi.1002859 (PMC3547803; doi:10.1371/journal.pcbi.1002859)
Supplement: Dataset S2 — Metabolite measurements for 6-week-old mice. This data is referred as 6 week data in the text and contains blood measurements for metabolites of DNL pathway. (DOC) [file pcbi.1002859.s002.doc]

**Dataset S2. Metabolite measurements for 6-week-old mice*.*** This data is referred as *6 week data* in the text and contains blood measurements for metabolites of DNL pathway.

| ID | Genotype | SEX | C10:0 | C12:0 | C14:0 | C16:1 | C16:0 | C18:1 | C18:0 | C18:2 (LA) | C18:3 (ALA) | C20:4 (AA) | C20:3 | C20:5 (EPA) | C22:6 (DHA) |
| --- | --- | --- | --- | --- | --- | --- | --- | --- | --- | --- | --- | --- | --- | --- | --- |
| 9416 | WT | F | 0.07938 | 0.14522 | 0.79128 | 0.16493 | 20.71592 | 2.70330 | 20.43675 | 2.63550 | 0.08199 | 0.07297 | 0.04240 | 0.00654 | 0.01135 |
| 9525 | WT | F | 0.07253 | 0.07644 | 0.35197 | 0.09860 | 18.40990 | 1.53112 | 20.65687 | 1.82250 | 0.04076 | 0.05192 | 0.02400 | 0.00282 | 0.00680 |
| 9389 | WT | F | 0.05920 | 0.10506 | 0.45697 | 0.14160 | 20.91039 | 2.30156 | 20.97855 | 2.35610 | 0.04948 | 0.07585 | 0.03343 | 0.00386 | 0.00913 |
| 9531 | WT | F | 0.07446 | 0.15490 | 0.80336 | 0.11513 | 22.46388 | 2.48754 | 22.29456 | 2.80868 | 0.07379 | 0.07241 | 0.02794 | 0.00361 | 0.01056 |
| 9532 | WT | F | 0.06697 | 0.10353 | 0.50010 | 0.14177 | 22.64656 | 2.21138 | 22.26171 | 2.61868 | 0.05588 | 0.09121 | 0.03092 | 0.00464 | 0.01159 |
| 9382 | WT | F | 0.07559 | 0.12440 | 0.59306 | 0.16432 | 23.44543 | 2.76241 | 22.96607 | 3.04424 | 0.06629 | 0.11179 | 0.04631 | 0.00498 | 0.01198 |
| 9291 | WT | M | 0.03813 | 0.12327 | 0.55029 | 0.21765 | 23.87623 | 2.74028 | 24.09046 | 2.97794 | 0.06868 | 0.05238 | 0.03484 | 0.00352 | 0.00837 |
| 9303 | WT | M | 0.06399 | 0.15914 | 0.77785 | 0.27280 | 23.86437 | 3.30192 | 21.66064 | 3.32849 | 0.08034 | 0.06416 | 0.04387 | 0.00475 | 0.01060 |
| 9317 | WT | M | 0.04604 | 0.13781 | 0.54671 | 0.19513 | 22.95195 | 2.45460 | 22.84566 | 2.95272 | 0.06593 | 0.05104 | 0.02995 | 0.00326 | 0.01100 |
| 9344 | WT | M | 0.08245 | 0.16265 | 0.50102 | 0.19779 | 22.11213 | 2.16416 | 21.13888 | 2.77729 | 0.05365 | 0.06918 | 0.03294 | 0.00579 | 0.01131 |
| 9348 | WT | M | 0.06659 | 0.14615 | 0.76125 | 0.27279 | 24.51175 | 3.58197 | 22.29189 | 3.78919 | 0.09735 | 0.06429 | 0.04082 | 0.00555 | 0.01167 |
| 9415 | CF | F | 0.07528 | 0.13832 | 0.82636 | 0.18816 | 24.04737 | 3.16929 | 23.10947 | 3.54779 | 0.10328 | 0.09773 | 0.04235 | 0.00902 | 0.01161 |
| 9562 | CF | F | 0.07241 | 0.11503 | 0.62375 | 0.18446 | 24.01737 | 2.87780 | 23.56966 | 3.22489 | 0.08075 | 0.11125 | 0.04751 | 0.00758 | 0.01285 |
| 9545 | CF | F | 0.06554 | 0.15732 | 0.41519 | 0.11726 | 20.78052 | 1.93960 | 21.72230 | 2.38843 | 0.05208 | 0.07987 | 0.03133 | 0.00378 | 0.00819 |
| 9414 | CF | F | 0.06234 | 0.11144 | 0.85998 | 0.15894 | 24.06028 | 3.21002 | 22.21739 | 3.32082 | 0.10186 | 0.07748 | 0.03339 | 0.00668 | 0.01012 |
| 9493 | CF | F | 0.05242 | 0.11667 | 0.78293 | 0.16046 | 24.26166 | 3.58429 | 23.83509 | 4.12665 | 0.11751 | 0.09982 | 0.03130 | 0.00568 | 0.01228 |
| 9426 | CF | F | 0.07734 | 0.11707 | 0.58692 | 0.12668 | 21.68145 | 2.24669 | 22.33603 | 2.82290 | 0.07621 | 0.07305 | 0.02520 | 0.00454 | 0.00864 |
| 9521 | CF | M | 0.09380 | 0.15810 | 0.79982 | 0.22676 | 28.89675 | 3.27954 | 27.82032 | 4.47055 | 0.11394 | 0.10344 | 0.04639 | 0.01043 | 0.01757 |
| 9547 | CF | M | 0.07236 | 0.15666 | 0.83311 | 0.23339 | 25.11885 | 3.30996 | 23.58631 | 4.01542 | 0.12116 | 0.09119 | 0.04396 | 0.00811 | 0.01555 |
