# Supplementary material for: ADEMA: An Algorithm to Determine Expected Metabolite Level Alterations Using Mutual Information
Source: PLoS Comput Biol. 2013 Jan 17;9(1):e1002859. doi: 10.1371/journal.pcbi.1002859 (PMC3547803; doi:10.1371/journal.pcbi.1002859)
Supplement: Dataset S3 — Liver profile for adult mice. This data is referred as liver profile in the text and contains blood measurements for metabolites of DNL pathway. (DOC) [file pcbi.1002859.s003.doc]

**Dataset S3. Liver profile for adult mice*.*** This data is referred as *liver profile* in the text and contains blood measurements for metabolites of DNL pathway.

| ID | genotype | Alanine | Glycine | Valine | Leucine | Isoleucine | Proline | Urea | Serine | Threonine | Aspartate | Methionine | glutamine | Oxo-proline | L-Phenylalanine | Tyrosine | Lactate | Glycerol | Succinate | Fumarate | β-alanine | Malate | PEP | alphaGP | Glucose | Citrate | pantothenic acid | uridine | inosine |
| --- | --- | --- | --- | --- | --- | --- | --- | --- | --- | --- | --- | --- | --- | --- | --- | --- | --- | --- | --- | --- | --- | --- | --- | --- | --- | --- | --- | --- | --- |
| 13548 | WT | 15.46965 | 0.82206 | 1.12962 | 4.92070 | 2.55153 | 2.21525 | 29.07082 | 1.57491 | 0.45899 | 1.34241 | 0.43545 | 3.32748 | 2.64131 | 0.88594 | 0.06638 | 36.11162 | 2.74232 | 11.93389 | 3.36660 | 0.11088 | 1.54469 | 0.39993 | 9.41774 | 373.46945 | 8.00072 | 0.12854 | 0.38882 | 1.67565 |
| 13550 | WT | 17.17362 | 0.96786 | 0.14918 | 4.72878 | 2.71502 | 2.74955 | 22.33761 | 1.24206 | 0.47166 | 0.51115 | 0.46282 | 2.40334 | 2.30319 | 0.83890 | 0.02233 | 34.97516 | 3.03051 | 11.75352 | 1.57772 | 0.16073 | 0.73118 | 0.44069 | 6.64756 | 432.61534 | 5.28372 | 0.07360 | 0.53828 | 1.10438 |
| 13553 | WT | 17.10162 | 0.76688 | 0.80109 | 3.27422 | 1.73199 | 1.95152 | 19.22493 | 0.81259 | 0.30320 | 0.61904 | 0.23256 | 2.20432 | 1.89073 | 0.60654 | 0.03930 | 37.07097 | 2.18012 | 13.30195 | 1.39079 | 0.09551 | 0.56768 | 0.50501 | 6.24420 | 195.39016 | 5.48333 | 0.07133 | 0.37555 | 1.79993 |
| 13556 | WT | 18.86267 | 0.91341 | 1.09572 | 4.51719 | 2.36334 | 2.39276 | 20.47616 | 1.10341 | 0.36381 | 0.83982 | 0.36084 | 2.88428 | 2.93514 | 0.75997 | 0.10834 | 34.17032 | 2.83636 | 14.27854 | 2.05912 | 0.09581 | 0.84684 | 1.10659 | 6.67539 | 275.86497 | 6.68786 | 0.11354 | 0.50677 | 2.13075 |
| 9095 | WT | 15.39915 | 0.97475 | 0.26515 | 3.92783 | 1.98456 | 2.38992 | 19.13865 | 1.52973 | 0.42856 | 0.64947 | 0.32876 | 2.10777 | 2.55656 | 0.59223 | 0.04107 | 19.37174 | 4.29664 | 9.34471 | 1.76126 | 0.15839 | 0.67695 | 0.81622 | 6.00798 | 156.27561 | 5.17370 | 0.06897 | 0.27411 | 1.45412 |
| 9111 | WT | 18.35613 | 0.97106 | 0.61997 | 4.51962 | 2.53061 | 2.45614 | 25.45497 | 1.59004 | 0.41461 | 0.91543 | 0.30337 | 3.29450 | 3.29611 | 0.76218 | 0.02748 | 34.84465 | 3.53879 | 12.22885 | 2.73248 | 0.23053 | 0.77038 | 1.16638 | 9.42431 | 233.38107 | 5.65723 | 0.07314 | 0.42985 | 3.18436 |
| 9172 | WT | 18.79872 | 0.82573 | 0.59077 | 3.84068 | 2.20949 | 2.52254 | 23.85422 | 1.49135 | 0.43957 | 0.82749 | 0.39048 | 2.57915 | 2.66015 | 0.64485 | 0.03068 | 29.92341 | 2.16319 | 10.38627 | 1.97961 | 0.61089 | 0.61829 | 0.82304 | 5.79268 | 289.68038 | 4.21766 | 0.07327 | 0.26378 | 1.78277 |
| 9173 | WT | 16.21149 | 0.89962 | 0.47911 | 3.67548 | 2.05299 | 2.07264 | 20.03214 | 1.34207 | 0.37662 | 0.70642 | 0.24936 | 2.07758 | 3.29303 | 0.56814 | 0.02372 | 21.42192 | 2.10939 | 10.85976 | 2.19524 | 0.35956 | 0.62385 | 0.74447 | 6.13307 | 237.29879 | 5.03748 | 0.10841 | 0.25470 | 1.35445 |
| 5643 | WT | 16.55557 | 1.07194 | 0.03609 | 4.28227 | 2.33904 | 2.40378 | 23.08895 | 1.37725 | 0.40804 | 0.69698 | 0.27423 | 1.62912 | 3.21201 | 0.65686 | 0.13677 | 22.84530 | 2.43095 | 12.19320 | 1.55517 | 0.27731 | 0.51423 | 0.87615 | 7.65279 | 272.62401 | 5.54726 | 0.06104 | 0.19422 | 2.58046 |
| 5644 | WT | 16.87877 | 0.82416 | 1.23443 | 4.84630 | 2.77870 | 2.51873 | 26.87584 | 1.31637 | 0.41972 | 0.84071 | 0.33255 | 2.49491 | 1.85578 | 0.70367 | 0.03843 | 23.92661 | 2.23899 | 11.76384 | 2.72175 | 0.22031 | 0.90591 | 0.91322 | 7.00684 | 298.88970 | 6.44989 | 0.09072 | 0.26416 | 1.42987 |
| 5645 | WT | 16.48589 | 0.49649 | 0.82731 | 3.24798 | 1.83918 | 2.04359 | 19.09393 | 1.18334 | 0.38004 | 0.80610 | 0.30473 | 2.53646 | 2.34884 | 0.55335 | 0.08936 | 27.83411 | 1.94427 | 12.55017 | 1.94487 | 1.03806 | 0.62026 | 0.39481 | 5.19678 | 135.79275 | 2.65366 | 0.06805 | 0.19035 | 1.14092 |
| 5646 | WT | 20.03704 | 1.03241 | 1.36538 | 5.54384 | 3.05793 | 3.01268 | 29.50916 | 1.59282 | 0.48946 | 1.64159 | 0.51233 | 4.32836 | 3.48270 | 0.84603 | 0.04435 | 48.95715 | 3.76316 | 20.71827 | 3.49555 | 0.66876 | 1.24741 | 1.40536 | 12.43250 | 336.77634 | 7.87114 | 0.13680 | 0.34515 | 2.47100 |
| 13499 | CF | 17.49852 | 1.40193 | 1.00147 | 4.36260 | 2.26042 | 3.01699 | 23.22047 | 1.63841 | 0.42648 | 0.93434 | 0.34724 | 2.32051 | 2.84336 | 0.73712 | 0.15251 | 38.16346 | 2.41507 | 11.69788 | 3.78624 | 0.11094 | 1.19915 | 0.65756 | 7.09933 | 144.42619 | 4.84029 | 0.10619 | 0.40890 | 2.11030 |
| 13500 | CF | 15.97597 | 1.16023 | 1.22342 | 4.55627 | 2.40238 | 2.94796 | 17.24462 | 1.11018 | 0.34224 | 0.99748 | 0.37580 | 3.58552 | 2.25936 | 0.78454 | 0.17956 | 21.33128 | 3.07548 | 13.32607 | 2.47432 | 0.11132 | 0.92027 | 0.54347 | 6.16416 | 143.86322 | 5.20908 | 0.12513 | 0.35476 | 1.48066 |
| 13609 | CF | 16.79435 | 0.88784 | 0.99855 | 3.71686 | 2.08571 | 2.45772 | 15.96338 | 1.21054 | 0.39179 | 0.31702 | 0.32433 | 2.21569 | 2.43620 | 0.65513 | 0.05712 | 23.09126 | 2.43333 | 12.06558 | 0.95384 | 0.23787 | 0.43093 | 1.14122 | 5.18691 | 192.27107 | 3.60914 | 0.06831 | 0.47698 | 1.58825 |
| 13611 | CF | 16.19293 | 0.92007 | 0.86316 | 3.61342 | 1.88425 | 2.07543 | 20.12832 | 1.12176 | 0.30808 | 0.71599 | 0.27304 | 2.33555 | 2.83282 | 0.57736 | 0.13154 | 27.95282 | 2.54304 | 11.10964 | 2.63168 | 0.16474 | 0.99905 | 0.62934 | 6.36548 | 226.45107 | 4.98815 | 0.06758 | 0.37192 | 2.50189 |
| 9136 | CF | 23.94939 | 1.16146 | 0.44138 | 3.77939 | 1.97954 | 3.25591 | 23.87325 | 1.29505 | 0.45117 | 0.65280 | 0.32181 | 3.54659 | 2.04712 | 0.59015 | 0.03816 | 30.41788 | 2.02097 | 12.26009 | 1.13537 | 0.58340 | 0.38361 | 0.90032 | 5.89067 | 191.59358 | 5.13013 | 0.07765 | 0.20067 | 0.54909 |
| 9144 | CF | 21.92624 | 1.05558 | 0.43113 | 3.33252 | 1.69280 | 2.57771 | 18.19055 | 1.49338 | 0.42794 | 0.62158 | 0.29766 | 1.80567 | 2.30282 | 0.51208 | 0.02474 | 19.28957 | 1.99532 | 8.60796 | 1.20795 | 0.41210 | 0.42222 | 0.89291 | 4.36400 | 131.40129 | 3.94818 | 0.04272 | 0.21161 | 0.68744 |
| 9231 | CF | 30.50666 | 1.37885 | 0.47265 | 4.66254 | 2.34068 | 3.99029 | 29.63932 | 1.65814 | 0.44683 | 1.46005 | 0.37978 | 3.68483 | 2.80511 | 0.72370 | 0.04636 | 54.19908 | 3.51391 | 15.83298 | 4.43363 | 0.89132 | 1.43397 | 1.08989 | 9.15675 | 270.09626 | 6.12311 | 0.11511 | 0.32692 | 2.64716 |
| 5629 | CF | 21.91857 | 0.82776 | 0.84803 | 3.07999 | 1.70180 | 2.71163 | 18.65580 | 1.08203 | 0.34870 | 0.60475 | 0.23644 | 1.94963 | 2.19499 | 0.45905 | 0.02344 | 26.12781 | 1.81247 | 13.42524 | 1.34014 | 0.67706 | 0.57371 | 0.87776 | 5.43946 | 133.35225 | 4.30262 | 0.07522 | 0.10289 | 0.73086 |
| 5681 | CF | 15.44679 | 0.88706 | 1.19725 | 4.89351 | 2.81761 | 2.48944 | 18.39627 | 1.38999 | 0.42938 | 0.71088 | 0.29166 | 1.87499 | 2.54787 | 0.72054 | 0.08867 | 19.72568 | 1.84045 | 12.21573 | 3.15680 | 0.18740 | 0.89550 | 0.98797 | 5.62188 | 153.15189 | 4.67749 | 0.03286 | 0.15390 | 2.23849 |
| 5683 | CF | 15.69396 | 0.71077 | 0.94998 | 4.03678 | 2.20411 | 2.12306 | 18.97201 | 1.07417 | 0.31941 | 0.69753 | 0.22945 | 1.85808 | 2.36811 | 0.58028 | 0.03034 | 20.47255 | 1.88652 | 11.08990 | 1.26419 | 0.46428 | 0.38722 | 0.86591 | 6.47936 | 167.76001 | 4.77898 | 0.06074 | 0.16531 | 2.52586 |
