# Supplementary material for: ADEMA: An Algorithm to Determine Expected Metabolite Level Alterations Using Mutual Information
Source: PLoS Comput Biol. 2013 Jan 17;9(1):e1002859. doi: 10.1371/journal.pcbi.1002859 (PMC3547803; doi:10.1371/journal.pcbi.1002859)
Supplement: Dataset S4 — In Silico Dataset generated using Wolf2000_Glycolytic_Oscillations Model. We have generated the following data by running 10 distinct simulations on Wolf2000_Glycolytic_Oscillations using different initial concentrations for Glucose. For each metabolite in each experiment we have obtained 75 values (there were 75 time points) and averaged them to obtain a representative value. We assumed variable group had higher (>10) initial Glucose concentrations and control group had low (<6) Glucose concentrations. (DOCX) [file pcbi.1002859.s004.docx]

| id | genotype | ATP | Glyceraldehyde 3-Phosphate+DHAP | Acetaldehyde | NAD | Pyruvate | Glucose | Extracellular acetaldehyde | 3PG | Fructose 1,6 Bisphosphate |
| --- | --- | --- | --- | --- | --- | --- | --- | --- | --- | --- |
| 1 | Variable | 2.166803765 | 0.564670427 | 0.076866115 | 0.59514903 | 8.365612809 | 1.267523608 | 0.024482642 | 0.696329591 | 5.141340485 |
| 2 | Variable | 2.231804261 | 0.561877761 | 0.076896605 | 0.596262309 | 8.357841406 | 1.275161087 | 0.024445484 | 0.698384227 | 5.109227535 |
| 3 | Variable | 2.23127388 | 0.558844866 | 0.077072974 | 0.598316349 | 8.343507137 | 1.277489364 | 0.02450019 | 0.698472127 | 5.091249294 |
| 7 | Variable | 2.217705702 | 0.563872533 | 0.077232515 | 0.597737771 | 8.351818738 | 1.335848513 | 0.024582673 | 0.71685571 | 5.096395451 |
| 8 | Variable | 2.223070581 | 0.567418744 | 0.077491617 | 0.599578278 | 8.340886517 | 1.362876547 | 0.024668637 | 0.739436251 | 5.085078327 |
| 9 | Control | 2.199282663 | 0.552804579 | 0.076251997 | 0.595631531 | 8.312401614 | 1.152826199 | 0.02426757 | 0.666805319 | 5.086979862 |
| 10 | Control | 2.219789524 | 0.555069062 | 0.076388467 | 0.596301524 | 8.315527888 | 1.167450406 | 0.024293298 | 0.674203433 | 5.113057517 |
| 4 | Control | 2.165452474 | 0.552799236 | 0.076003222 | 0.593660469 | 8.318447252 | 1.112943807 | 0.024194407 | 0.653443066 | 5.112978504 |
| 5 | Control | 2.176107037 | 0.554125884 | 0.075918348 | 0.593440461 | 8.314586631 | 1.090137156 | 0.024152418 | 0.655111545 | 5.106177894 |
| 6 | Control | 2.168791746 | 0.552597061 | 0.075904288 | 0.593816102 | 8.306569743 | 1.082356378 | 0.024157516 | 0.654235306 | 5.104658218 |

**Dataset S4. In Silico Dataset generated using Wolf2000_Glycolytic_Oscillations Model*.*** We have generated the following data by running 10 distinct simulations on Wolf2000_Glycolytic_Oscillations using different initial concentrations for Glucose. For each metabolite in each experiment we have obtained 75 values (there were 75 time points) and averaged them to obtain a representative value. We assumed variable group had higher (>10) initial Glucose concentrations and control group had low (<6) Glucose concentrations.
