# Supplementary figures and images for: ADEMA: An Algorithm to Determine Expected Metabolite Level Alterations Using Mutual Information
Source: PLoS Comput Biol. 2013 Jan 17;9(1):e1002859. doi: 10.1371/journal.pcbi.1002859 (PMC3547803; doi:10.1371/journal.pcbi.1002859)

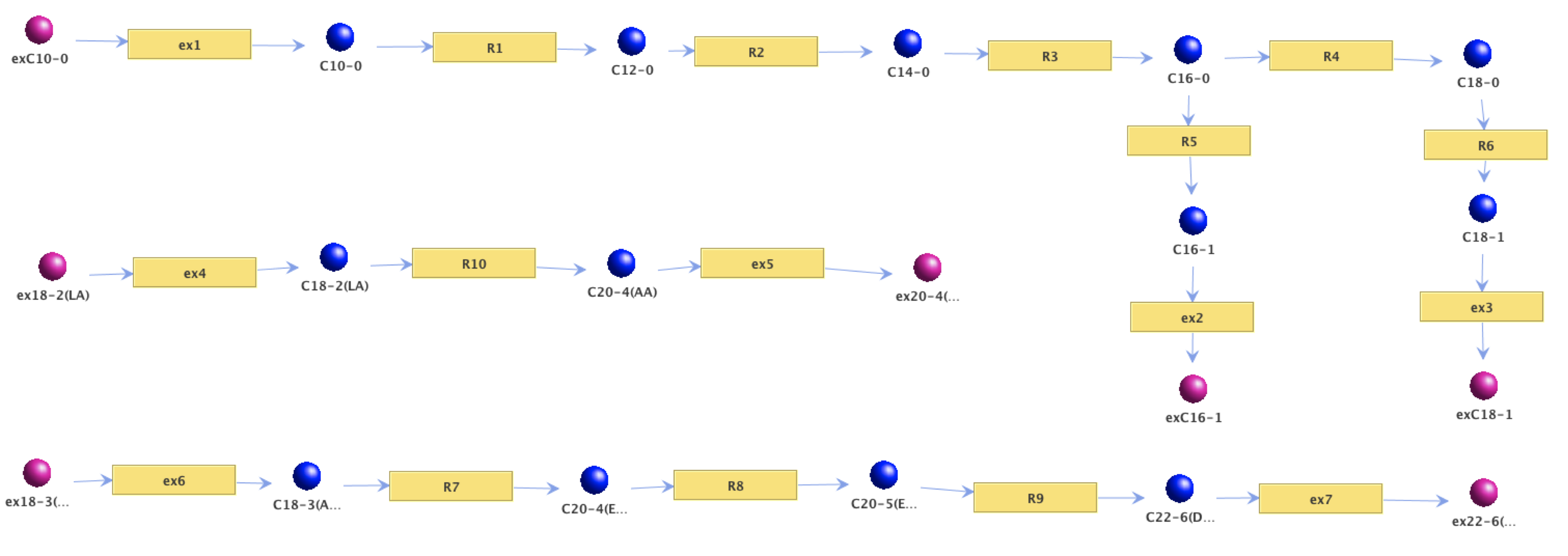

Supplement: Figure S1 — YANA screenshot of the network created to obtain EFMs for Dataset S1 and Dataset S2. In this figure blue circles represent internal metabolites and pink circles represent external metabolites. External metabolites are not considered in the analysis, but they are input to specify the entrance and exit points to the network. Rectangles represent reactions that relate metabolites. These reactions are “abstract” reactions that might contain one or more reactions. This network represents the DNL pathway and was used to obtain the EFMs. (DOC) [file pcbi.1002859.s005.doc]

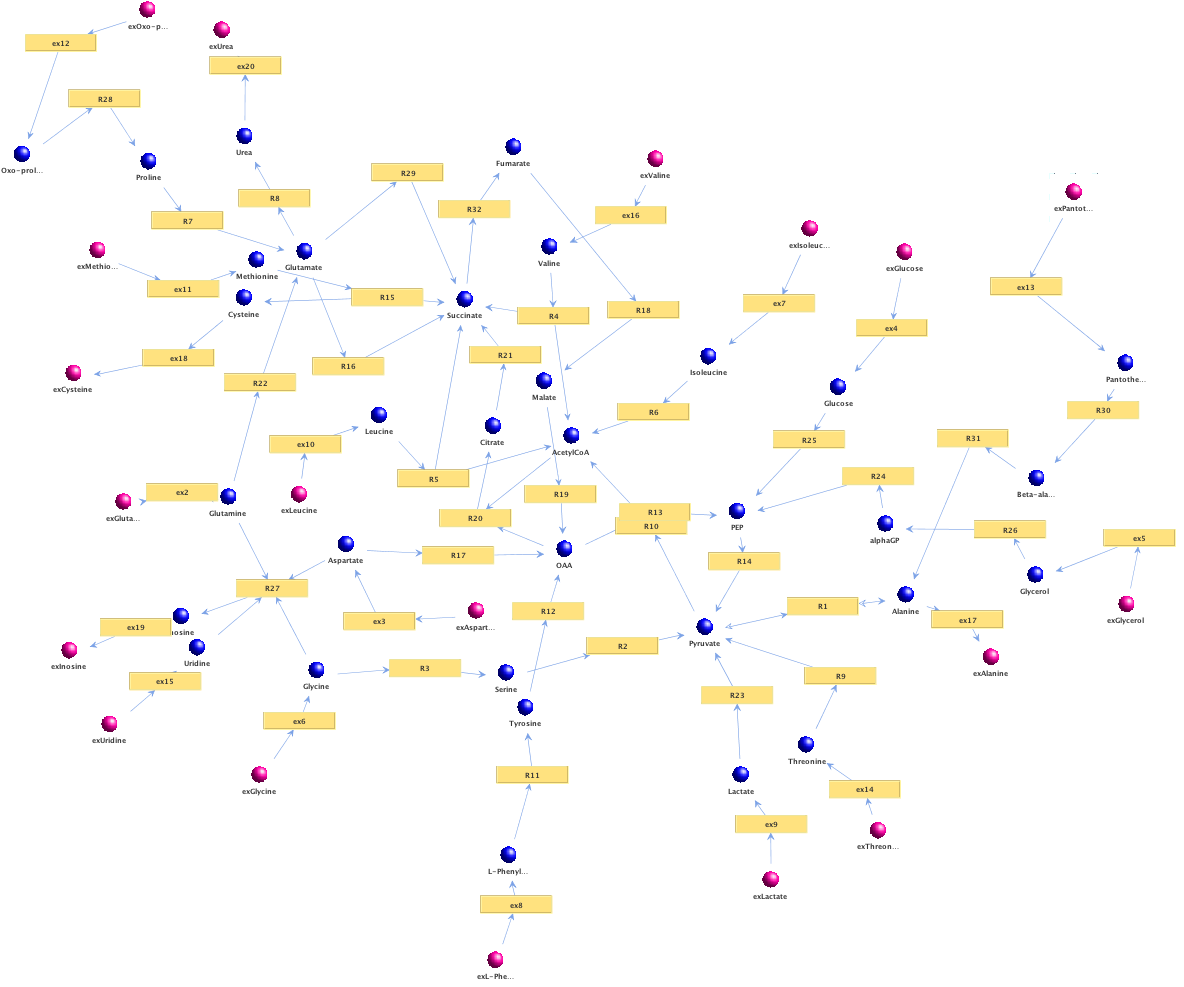

Supplement: Figure S2 — YANA screenshot of the network created to obtain EFMs for Dataset S3. Colors and shapes representing entities are same as in Figure S1. This network is formed by linking related metabolites together according to Selway et al. [51] and was used to obtain EFMs. (DOC) [file pcbi.1002859.s006.doc]

**
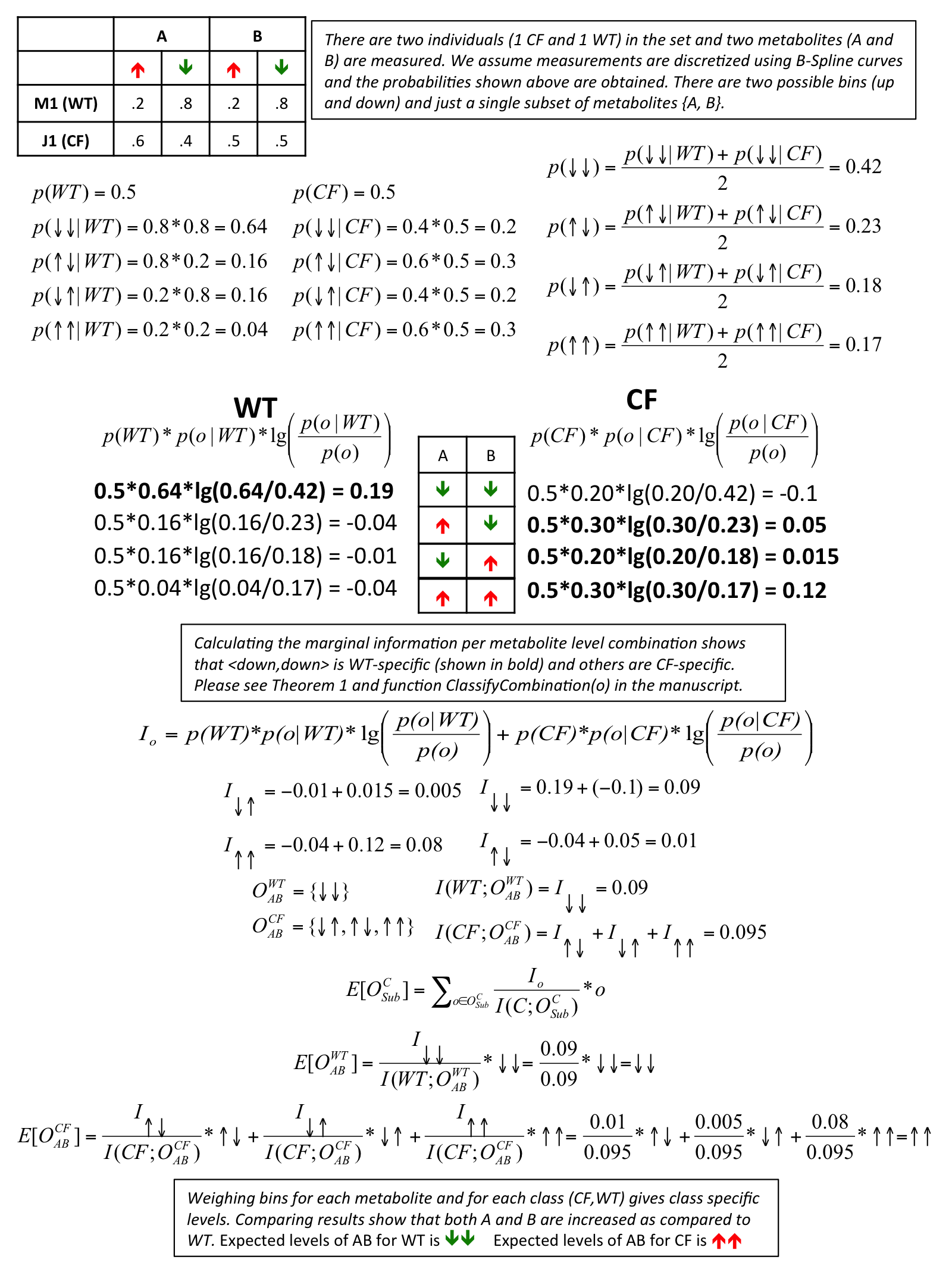
**

Supplement: Figure S3 — Example that shows basic calculations done for ADEMA. Given one individual per class and two measured metabolites, ADEMA generates 4 possible metabolite combinations and based on the probabilities obtained using B-spline curves (in this case estimates) expected levels per group are found. ADEMA first classifies bin combinations as WT- and CF-specific to conclude that ↑↑ are the expected levels for CF and ↓↓ are the expected levels for WT. (DOC) [file pcbi.1002859.s007.doc]
