# Supplementary material for: ADEMA: An Algorithm to Determine Expected Metabolite Level Alterations Using Mutual Information
Source: PLoS Comput Biol. 2013 Jan 17;9(1):e1002859. doi: 10.1371/journal.pcbi.1002859 (PMC3547803; doi:10.1371/journal.pcbi.1002859)
Supplement: Table S1 — Accuracy results for different M,k and max subset size parameters for Dataset S1. (DOC) [file pcbi.1002859.s008.doc]

**Table S1. Accuracy results for different *M,k and max subset size* parameters for Dataset S1.**

|  |  | M=3 | | M=4 | | M=5 | | M=6 | |
| --- | --- | --- | --- | --- | --- | --- | --- | --- | --- |
|  |  | k=2 | k=3 | k=2 | k=3 | k=2 | k=3 | k=2 | k=3 |
| Max Subset Size | 2 | 0.5625 | 0.5625 | 0.5625 | 0.5625 | 0.6875 | 0.6875 | 0.6875 | 0.6875 |
|  | 3 | 0.8125 | 0.5625 | 0.875 | 0.8125 | 0.9375 | 1 | 0.9375 | 0.9375 |
|  | 4 | 0.8125 | 0.5625 | 0.9375 | 0.875 | 0.9375 | 1 | 0.9375 | 0.9375 |
|  | 5 | 0.8125 | 0.5625 | 0.9375 | 0.8125 | 0.9375 | 1 | 0.9375 | 1 |
|  | 6 | 0.8125 | 0.625 | 0.9375 | 0.875 | 1 | 1 | 0.9375 | 1 |
|  | 7 | 0.8125 | 0.625 | 0.9375 | 0.875 | 1 | 1 | 1 | 1 |
