# Supplementary material for: ADEMA: An Algorithm to Determine Expected Metabolite Level Alterations Using Mutual Information
Source: PLoS Comput Biol. 2013 Jan 17;9(1):e1002859. doi: 10.1371/journal.pcbi.1002859 (PMC3547803; doi:10.1371/journal.pcbi.1002859)
Supplement: Table S2 — Accuracy results for different M,k and max subset size parameters for Dataset S2. Best result is marked as bold. (DOC) [file pcbi.1002859.s009.doc]

**Table S2. Accuracy results for different *M,k and max subset size* parameters for Dataset S2. Best result is marked as bold.**

|  |  | M=3 | | M=4 | | M=5 | | M=6 | |
| --- | --- | --- | --- | --- | --- | --- | --- | --- | --- |
|  |  | k=2 | k=3 | k=2 | k=3 | k=2 | k=3 | k=2 | k=3 |
| Max Subset Size | 2 | 0.736842105 | 0.736842105 | 0.789473684 | 0.789473684 | 0.684210526 | 0.736842105 | 0.736842105 | 0.789473684 |
|  | 3 | 0.736842105 | 0.736842105 | 0.789473684 | 0.684210526 | 0.789473684 | 0.789473684 | 0.789473684 | 0.684210526 |
|  | 4 | 0.684210526 | 0.736842105 | 0.842105263 | 0.736842105 | 0.789473684 | 0.789473684 | 0.789473684 | 0.736842105 |
|  | 5 | 0.684210526 | 0.631578947 | 0.631578947 | 0.473684211 | 0.631578947 | 0.578947368 | 0.631578947 | 0.631578947 |
|  | 6 | 0.736842105 | 0.789473684 | 0.684210526 | 0.631578947 | 0.736842105 | 0.736842105 | 0.684210526 | 0.684210526 |
|  | 7 | 0.789473684 | **0.842105263** | 0.789473684 | 0.789473684 | 0.736842105 | 0.789473684 | 0.631578947 | 0.789473684 |
