# Supplementary material for: ADEMA: An Algorithm to Determine Expected Metabolite Level Alterations Using Mutual Information
Source: PLoS Comput Biol. 2013 Jan 17;9(1):e1002859. doi: 10.1371/journal.pcbi.1002859 (PMC3547803; doi:10.1371/journal.pcbi.1002859)
Supplement: Table S3 — Time results (secs) for different M,k and max subset size parameters for Dataset S2. (DOC) [file pcbi.1002859.s010.doc]

**Table S4. Time results for different *M,k and max subset size* parameters for Dataset S1.**

|  |  | M=3 | | M=4 | | M=5 | | M=6 | |
| --- | --- | --- | --- | --- | --- | --- | --- | --- | --- |
|  |  | k=2 | k=3 | k=2 | k=3 | k=2 | k=3 | k=2 | k=3 |
| Max Subset Size | 2 | 0.021125 | 0.01125 | 0.0085 | 0.010375 | 0.012125 | 0.010875 | 0.01275 | 0.01975 |
|  | 3 | 0.0158125 | 0.0144375 | 0.0086875 | 0.009 | 0.01525 | 0.01175 | 0.0196875 | 0.022125 |
|  | 4 | 0.0123125 | 0.013375 | 0.009375 | 0.0100625 | 0.0195625 | 0.0143125 | 0.02475 | 0.0295625 |
|  | 5 | 0.0140625 | 0.0161875 | 0.013 | 0.0169375 | 0.02975 | 0.033375 | 0.0470625 | 0.068625 |
|  | 6 | 0.0204375 | 0.025875 | 0.033125 | 0.0731875 | 0.104 | 0.245875 | 0.2784375 | 0.8625625 |
|  | 7 | 0.0403125 | 0.0766875 | 0.15825 | 0.6589375 | 0.868375 | 3.5903125 | 3.0074375 | 15.494125 |
