# Supplementary material for: ADEMA: An Algorithm to Determine Expected Metabolite Level Alterations Using Mutual Information
Source: PLoS Comput Biol. 2013 Jan 17;9(1):e1002859. doi: 10.1371/journal.pcbi.1002859 (PMC3547803; doi:10.1371/journal.pcbi.1002859)
Supplement: Table S4 — Time results (secs) for different M,k and max subset size parameters for Dataset S2. (DOC) [file pcbi.1002859.s011.doc]

**Table S5. Time results (secs) for different *M,k and max subset size* parameters for Dataset S2.**

|  |  | M=3 | | M=4 | | M=5 | | M=6 | |
| --- | --- | --- | --- | --- | --- | --- | --- | --- | --- |
|  |  | k=2 | k=3 | k=2 | k=3 | k=2 | k=3 | k=2 | k=3 |
| Max Subset Size | 2 | 0.015894737 | 0.006736842 | 0.007157895 | 0.007421053 | 0.011789474 | 0.015473684 | 0.014789474 | 0.012842105 |
|  | 3 | 0.007 | 0.007578947 | 0.007526316 | 0.007894737 | 0.016105263 | 0.016947368 | 0.02 | 0.021473684 |
|  | 4 | 0.006631579 | 0.007473684 | 0.008315789 | 0.009368421 | 0.018947368 | 0.021789474 | 0.024 | 0.028368421 |
|  | 5 | 0.007578947 | 0.008684211 | 0.011894737 | 0.017263158 | 0.033105263 | 0.043 | 0.051526316 | 0.069684211 |
|  | 6 | 0.011736842 | 0.014421053 | 0.035789474 | 0.070473684 | 0.107894737 | 0.308578947 | 0.293315789 | 1.008368421 |
|  | 7 | 0.034210526 | 0.051789474 | 0.205947368 | 0.710578947 | 1.081105263 | 5.309631579 | 3.653789474 | 22.15363158 |
