# Supplementary material for: ADEMA: An Algorithm to Determine Expected Metabolite Level Alterations Using Mutual Information
Source: PLoS Comput Biol. 2013 Jan 17;9(1):e1002859. doi: 10.1371/journal.pcbi.1002859 (PMC3547803; doi:10.1371/journal.pcbi.1002859)
Supplement: Text S1 — Proof for Theorem 1. (DOC) [file pcbi.1002859.s012.doc]

**Text S1. Proof for Theorem 1.**
